# A Bootstrap Based Measure Robust to the Choice of Normalization Methods for Detecting Rhythmic Features in High Dimensional Data

**DOI:** 10.3389/fgene.2018.00024

**Published:** 2018-02-02

**Authors:** Yolanda Larriba, Cristina Rueda, Miguel A. Fernández, Shyamal D. Peddada

**Affiliations:** ^1^Departamento de Estadística e Investigación Operativa, Universidad de Valladolid, Valladolid, Spain; ^2^Biostatistics and Computational Biology Branch, National Institute of Environmental Health Sciences, Durham, NC, United States; ^3^Department of Biostatistics, University of Pittsburgh, Pittsburgh, PA, United States

**Keywords:** rhythmicity, high-throughput technologies, normalization, oscillatory systems, circadian genes

## Abstract

**Motivation:** Gene-expression data obtained from high throughput technologies are subject to various sources of noise and accordingly the raw data are pre-processed before formally analyzed. Normalization of the data is a key pre-processing step, since it removes systematic variations across arrays. There are numerous normalization methods available in the literature. Based on our experience, in the context of oscillatory systems, such as cell-cycle, circadian clock, etc., the choice of the normalization method may substantially impact the determination of a gene to be rhythmic. Thus rhythmicity of a gene can purely be an artifact of how the data were normalized. Since the determination of rhythmic genes is an important component of modern toxicological and pharmacological studies, it is important to determine truly rhythmic genes that are robust to the choice of a normalization method.

**Results:** In this paper we introduce a rhythmicity measure and a bootstrap methodology to detect rhythmic genes in an oscillatory system. Although the proposed methodology can be used for any high-throughput gene expression data, in this paper we illustrate the proposed methodology using several publicly available circadian clock microarray gene-expression datasets. We demonstrate that the choice of normalization method has very little effect on the proposed methodology. Specifically, for any pair of normalization methods considered in this paper, the resulting values of the rhythmicity measure are highly correlated. Thus it suggests that the proposed measure is robust to the choice of a normalization method. Consequently, the rhythmicity of a gene is potentially not a mere artifact of the normalization method used. Lastly, as demonstrated in the paper, the proposed bootstrap methodology can also be used for simulating data for genes participating in an oscillatory system using a reference dataset.

**Availability:** A user friendly code implemented in R language can be downloaded from http://www.eio.uva.es/~miguel/robustdetectionprocedure.html

## 1. Introduction

One of the major difficulties dealing with high-throughput gene-expression experiments is the noisy nature of the data (Tu et al., 2002; Klebanov and Yakovlev, 2007) that is intrinsic to each array. Thus an important component of gene-expression analysis is pre-processing the data to remove (or reduce) sources of variation of non-biological origin among arrays (Bolstad et al., 2003; Irizarry et al., 2003a). A variety of pre-processing methods are available in literature, such as the Model-based Expression Index (MBEI) (Li and Wong, 2001), MAS 5.0 (Hubbell et al., 2002; Liu et al., 2003), and Robust Multi-array Average (RMA) (Irizarry et al., 2003b). They usually involve three distinct steps, namely, Background correction, Normalization, and Summarization (Wu, 2009). Normalization is an important component of pre-processing (Bolstad et al., 2003; Cheng et al., 2016), since it removes technical (i.e., non-biological) variations from the expression data. There are numerous methods available in the literature to normalize gene expression data and in this paper we consider the following popular normalization methods: *Quantile* (Bolstad et al., 2003), *(Cyclic) Loess* (Bolstad et al., 2003), *Contrast* (Astrand, 2003), *Constant* (Bolstad et al., 2003), *Invariant Set* (Li and Wong, 2001), *Qspline* (Workman et al., 2002), and *Variance Stabilization Normalization (VSN)* (Huber et al., 2002). Each normalization strategy is based on certain model and assumptions. Consequently, the resulting normalized expression data, and the downstream analyses, are expected to depend upon the normalization method used. It is well-known that many biological processes, such as metabolic cycle (Slavov et al., 2012), cell-cycle (Rustici et al., 2004; Oliva et al., 2005; Peng et al., 2005; Barragán et al., 2015) or the circadian clock (Hughes et al., 2009) are governed by oscillatory systems consisting of numerous components that exhibit rhythmic or periodic patterns over time. There are several algorithms available in the literature to determine whether a gene is rhythmic or not. Some recent examples include JTK_Cycle (from now on JTK) (Hughes et al., 2010), RAIN (Thaben and Westermark, 2014), and ORIOS (Larriba et al., 2016). The performance of such algorithms potentially depends upon, among other factors, the normalization methods used. For example, Rustici et al. (2004); Oliva et al. (2005); Peng et al. (2005) conducted long-series time-course cell-cycle microarray study on *Schizosaccharomyces pombe* to identify rhythmic genes. The number of such genes identified by the three studies vary. Oliva et al. (2005) discovered 750 genes to be rhythmic, Peng et al. (2005) found about 747 rhythmic genes, whereas Rustici et al. (2004) discovered only 407 rhythmic genes. What is more interesting is that only 150 genes were identified to be periodic by all three studies. For more details, one may refer to Caretta-Cartozo et al. (2007).

There has not been a systematic evaluation of the impact of normalization methods on identifying rhythmic genes in studies involving oscillatory systems. Yet, researchers are interested in identifying rhythmic genes. A goal of this paper is to introduce a bootstrap based rhythmicity measure that is highly correlated across various normalization methods. As a consequence, a gene declared to be rhythmic under one normalization scheme is likely to be rhythmic under a different one. A by-product of our methodology is that the bootstrap procedure introduced in this paper can be used for simulating potentially realistic time-course circadian gene-expression data. Although several authors have developed algorithms for simulating time-course gene-expression data (cf. Freudenberg et al., 2004; Nykter et al., 2006; Parrish et al., 2009; Dembélé, 2013), each of them was specific to the experiment discussed in the paper and not broadly applicable. However, our proposed algorithm is very generic. It not only helps to identify rhythmic genes, but it also provides a tool to simulate potentially realistic circadian gene-expression data.

## 2. Methods

**Figure 1:**
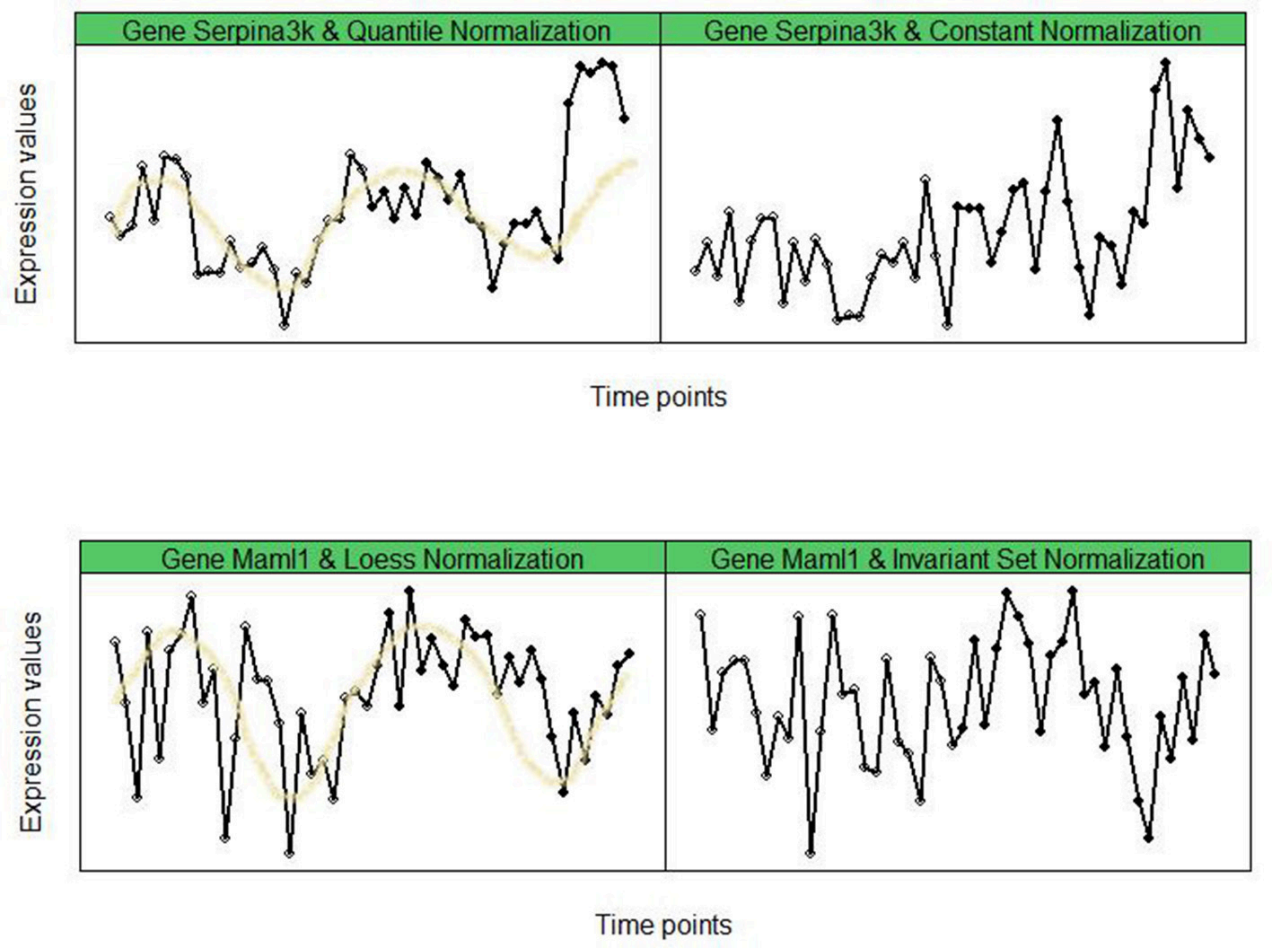
Time-course gene-expression for genes *Serpina3k* **(top)** and *Maml1* **(bottom)**. *Serpina3k* and *Maml1* are identified as rhythmic by ORIOS according to *Quantile* and *Loess* normalizations, respectively. But they are identify as non-rhythmic by ORIOS for *Constant* and *Invariant Set* normalizations, respectively.

We begin this section by considering time-course data on two genes, namely, *Serpina3k* and *Maml1* from mouse liver tissue (see Hughes et al., 2009) as the motivating examples. We normalized the data using, *Quantile, Constant, (Cyclic) Loess*, and *Invariant Set* normalization methods. For illustration purposes, in the top panel of Figure 1 we report the time-course plots of the gene *Serpina3k* using *Quantile* (left panel) and *Constant* (right panel) normalization procedures. In the bottom panel of Figure 1 we provide the time-course plots of the gene *Maml1* using *Loess* (left panel) and *Invariant Set* (right panel) normalization procedures. As one can see, the time-course profiles of these genes are markedly different, depending upon which normalization procedure was used. Furthermore, if rhythmicity detection algorithm ORIOS is used then *Serpina3k* and *Maml1* are rhythmic genes if *Quantile* and *Loess* normalizations are used, respectively. But they cease to be rhythmic genes if *Constant* and *Invariant Set* normalization procedures are used. Similar conclusions are drawn if other rhythmicity detection algorithms, such as JTK and RAIN are used on these data. Such results in a genome-wide analysis can be very confusing and difficult to interpret.

Given a normalization method *n* and a rhythmicity detection algorithm *a*, the identification of rhythmic genes is based on the Benjamini-Hochberg adjusted *p*-values [*p*-value^*g*^ (*n, a*), for*g* = 1, …, *G*]. For each gene *g* = 1, …, *G* we define the standard measure of gene rhythmicity associated to gene *g*, as follows:

(1) $$\begin{array}{r} M^{g}\left(n,a\right)=1-{\text{p}-\text{value}}^{g}\left(n,a\right). \end{array}$$

In a vector notation we write **M**(*n, a*) = [*M*^1^(*n, a*), …, *M*^*G*^(*n, a*)], whose components take values between 0 and 1. Closer 0 indicates potentially non-rhythmic gene and closer 1 indicates potentially rhythmic gene.

For the plots in Figure 1 we have *M*^*Serpina*3*k*^(*Quantile, ORIOS*) = 0.996, *M*^*Serpina*3*k*^ (*Constant, ORIOS*) = 0.639, *M*^*Maml*1^(*Loess, ORIOS*) = 0.992, and *M*^*Maml*1^(*InvariantSet*, *ORIOS*) = 0.668. Thus implying *Serpina3k* is potentially rhythmic under *Quantile* normalization but not under *Constant* and similarly, *Maml1* potentially rhythmic under *Loess* normalization but not likely under *Invariant Set*. This observation that normalization method *n* may impact the rhythmicity of a gene is not limited to the above genes but is rather a common feature of long-series time-course data as noted in Table 1. Of course, as seen in Table 1, the rhythmicity algorithm *a* may also impact on determining if a gene is rhythmic or not. In modern pharmacological and toxicological studies (Zhang et al., 2014), there is a need for objective determination of rhythmic genes using high-throughput time-course gene-expression data. Motivated by this, we now introduce **M**_*Robust*_(*n, a*), a modification of **M**(*n, a*) which is more robust with respect to *n*, the normalization method, than **M**(*n, a*) is. The proposed bootstrap methodology also provides us a tool to simulate time-course expression data for genes participating in oscillatory systems such as the circadian clock using a reference dataset.

**Table 1:** Number of genes in mouse liver (Hughes et al., 2009) detected as rhythmic by ORIOS, JTK, and RAIN according to the different normalization strategies and *M*^*g*^(*n, a*) ≥ 0.99 for *g* = 1, …, 45, 101.

**Normalization strategy**	**ORIOS**	**JTK**	**RAIN**
0 Unnormalized	6,432	923	4,196
1 Quantile	9,259	4,998	12,381
2 Loess	8,812	3,932	10,571
3 Contrast	8,435	4,181	10,273
4 Constant	6,657	2,726	9,357
5 Invariant set	9,604	5,062	13,385
6 Qspline	9,163	4,546	11,828
7 VSN	8,397	3,608	10,700

### 2.1. Bootstrap methodology

Let **R** denote the tri-dimensional array of raw intensities obtained from a reference high-throughput data of an oscillatory system, such as the circadian clock. Data in **R** are expressed at probe level, where $R_{pt}^{g}$ states the raw intensity value for gene *g* on probe *p* at time point (*array*) *t*, where *g* = 1, …, *G*, *p* = 1, …, *P*, and *t* = 1, …, *T*. Let **X** be the tri-dimensional array derived from **R** after background correction. After normalization and summarization steps, a matrix of gene-expression values is finally obtained as the output of the pre-processing (see Figure S1 in the Supplementary Material for details). The bootstrap approach proposed in this work is based on a linear model from corrected intensities **X** of a reference dataset as follows. Let *b* = 1, …, *B*, denote bootstrap replications. Simulated gene-expression datasets **X**^(*b*)*^ are generated according to parametric bootstrap, see Efron and Tibshirani (1994), as:

(2) $$\begin{array}{r} \log_{2}\left(X_{pt}^{\left(b\right)g*}\right)={\hat{\alpha }}_{p}^{g}+{\hat{\beta }}_{t}^{g}+\epsilon_{pt}^{\left(b\right)g*}, \end{array}$$

where *g* = 1, …, *G*, *p* = 1, …, *P*, *t* = 1, …, *T*, *b* = 1, …, *B*, and ${\left\{{\hat{\alpha }}_{p}^{g}\right\}}_{g=1}^{G}$ and ${\left\{{\hat{\beta }}_{t}^{g}\right\}}_{g=1}^{G}$ denote original estimates of probe and array effects obtained from corrected (and unnormalized) intensities **X**. Following the methodology described in Irizarry et al. (2003b), the median polish algorithm is used to estimate model parameters (Emerson and Hoaglin, 1983). This algorithm is similar to a two-way ANOVA based estimation procedure except that it employs medians instead of means to ensure robustness to outliers. Additionally as explained in Irizarry et al. (2003b), it allows taking into account probe and array effects. $\epsilon_{pt}^{\left(b\right)g*}$ are identically and independently distributed according to a normal distribution $N\left(0,{\hat{\sigma }}^{2g}\right),$ where ${\hat{\sigma }}^{2g}$ is the usual *MSE* under the original two-way model. From Equation (2) it is important to recognize that we are bootstrapping the residuals while centering the bootstrap data (log_2_(**X**)) at the true observed signal. Thus the mean signal over the bootstrap samples retains the original expression and hence there is no loss of information in the mean signal through bootstrapping. If the expression data are count data, as in the case of RNA-seq, the observed counts may be transformed using a suitable variance stabilization transformation before appealing to the above model. It is common to model RNA-seq data either using standard Poisson or Poisson with extra-variability in the Poisson parameter by using a gamma prior which leads to modeling the RNA-seq data using a negative binomial distribution. In both cases the variance stabilizing transformation is known from the literature, which are either square transformation (for Poisson) or arc sinh square transformation (for negative binomial), see Guan (2009).

**Figure 2:**
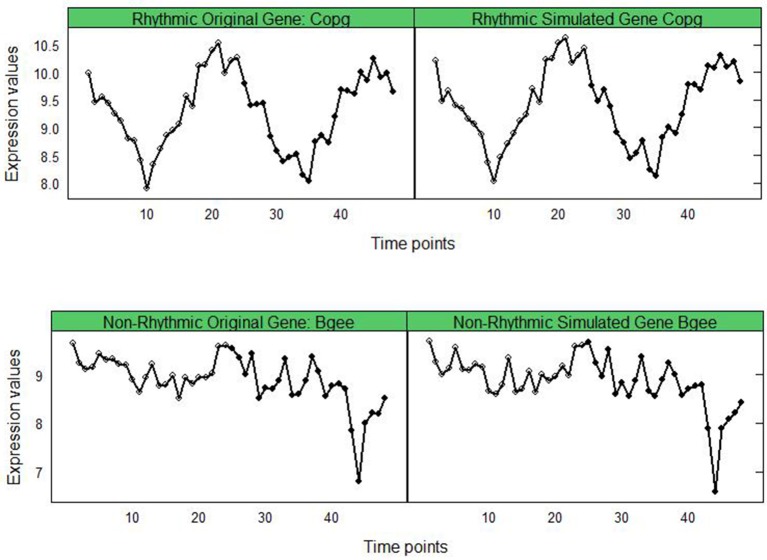
Original vs. Simulated gene-expression for genes *Copg* **(top)** and *Bgee* **(bottom)** showing the effect of bootstrapping. **(Left)** Corrected (and unnormalized) gene-expression from the reference dataset (*mouse liver tissue*). **(Right)** Simulated gene-expression attained after bootstrapping.

Using the time-course gene-expression data on *Copg* and *Bgee* (top and bottom left panels in Figure 2, respectively), we demonstrate how well our bootstrap based simulated data (the two right panels in Figure 2) resembles the pattern of expression of the real data. Thus it suggests that, in addition to detecting rhythmic genes robustly, our bootstrap methodology may also be useful for simulating reasonably realistic time-course expression patterns.

### 2.2. Robust measure of gene rhythmicity

For a rhythmicity detection algorithm *a* and a normalization strategy *n*, and a random realization of data, consider the rhythmicity statistic **M**(*n, a*). Let **θ**(*n, a*) = 𝔼(**M**(*n, a*)) be the parameter of interest and $\hat{\theta }\left(n,a\right)=\text{M}\left(n,a\right)$ be its estimator. For the *b*^*th*^ bootstrap sample using Equation (2), *b* = 1, 2, …, *B*, let ${\hat{\theta }}^{\left(b\right)*}\left(n,a\right)=\left({\hat{\theta }}^{1\left(b\right)*}\left(n,a\right),\ldots ,{\hat{\theta }}^{G\left(b\right)*}\left(n,a\right)\right),$ denote the bootstrap estimate of **θ**(*n, a*). Let $\hat{𝔼}\left(\hat{\theta }\left(n,a\right)\right)=\frac{1}{B}\overset{B}{\underset{b=1}{\sum }}\left({\hat{\theta }}^{\left(b\right)*}\left(n,a\right)\right)$ and $\hat{RMS𝔼}\left(\hat{\theta }\left(n,a\right)\right)=\sqrt{\frac{1}{B-1}\overset{B}{\underset{b=1}{\sum }}{\left({\hat{\theta }}^{\left(b\right)*}\left(n,a\right)-\hat{\theta }\left(n,a\right)\right)}^{2}}.$ Then we define

(3) $$\begin{array}{r} M_{\text{Robust}}\left(n,a\right)=\hat{E}\left(\hat{\theta }\left(n,a\right)\right)-\hat{RMSE}\left(\hat{\theta }\left(n,a\right)\right), \end{array}$$

as measure of gene rhythmicity. We call it a “robust” measure of gene rhythmicity because, as demonstrated later in this paper, by correcting for sample to sample variation in the rhythmicity measure (i.e., *RMSE*), it reduces the effect of the normalization method used.

## 3. Results

We re-analyzed three publicly available datasets (http://www.ncbi.nlm.nih.gov/geo/) of Hughes et al. (2009), the mouse liver (GSE11923) and mouse pituitary data and the NIH3T3 mouse cell line data (GSE11922). Due to space limitations, and since similar results were obtained in the three cases, we only report the results for the mouse liver data in the main paper and defer the rest of the results to the Supplementary Material document. The mouse liver data consisted of 45,101 probe sets (genes) at 48 time points representing two periods. Taking *M*^*g*^(*n, a*) ≥ 0.99 as the criterion to declare a gene to be rhythmic (the choice of this criterion is motivated by the findings of Larriba et al., 2016), in Table 1 we summarize the results of three rhythmicity detection algorithms, namely ORIOS, JTK, and RAIN using unnormalized data and seven normalization methods (*0.-Unnormalized, 1.-Quantile, 2.-(Cyclic) Loess, 3.-Contrast, 4.-Constant, 5.-Invariant Set, 6.-Qspline, 7.-VSN*). The number of rhythmic genes identified varies vastly among the normalization methods within each rhythmicity detection algorithm (Table 1). Thus it suggests that normalization methods have a large influence on whether a gene is classified as rhythmic or not.

To better illustrate this fact, a multiple correspondence analysis (MCA) was performed. MCA is an extension of correspondence analysis which allows one to analyze the pattern of relationships among several categorical variables (Benzécri, 1979; Greenacre, 1984). Since we consider three rhythmicity identification algorithms and eight normalization strategies consisting of unnormalized data and 7 normalization methods, each probe set can be described by 24 binary variables consisting of 1′s and 0′s depending on whether an algorithm *a* and a normalization strategy *n* declare a gene to be rhythmic or not. Thus resulting in a matrix of 45,101 rows and 24 columns.

**Figure 3:**
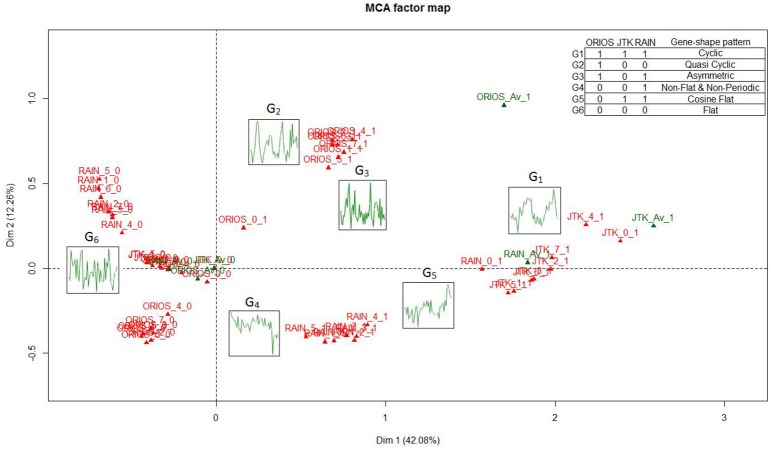
Multiple Correspondence Analysis factor map for the different gene profiles under all normalizations and algorithms considered, together with the averaged rhythmic and non-rhythmic profiles for each algorithm and the six gene patterns defined in the table. The Figure exposes the relationship between the first axis and rhythmicity (with rhythmic genes on the right hand side), and how the second axis separates the different detection algorithms.

MCA is a dimension reduction procedure that can be used to represent distances among high dimensional vectors in a low-dimensional space, such as 2-dimensional plane. Using the MCA plots, one typically tries to interpret what each axis represents and evaluates relationships among the categories of different variables based on the distance among their representations on the graph. The MCA plot based on the first two dimensions, which explain ~54% of the total variation in the data, is provided in Figure 3. Elements of the plot are as follows. For a rhythmicity algorithm *a*, a normalization method *n* and a rhythmicity category *r* (*r* = 1 if genes are declared as rhythmic and *r* = 0 if genes are declared as non-rhythmic), we plotted 3 × 8 × 2 categories denoted by *a*_*n*_*r*. Then, we averaged the expression values of those genes that are declared as rhythmic (or non-rhythmic) under all normalizations strategies, i.e., those with *r* = 1 (or *r* = 0) for all strategies under a given algorithm, and overlaid these averaged profiles on the plot. For algorithm *a*, the averaged profile of rhythmic genes is denoted by *a*_*Av*_1 and the averaged profile of non-rhythmic one is denoted by *a*_*Av*_0. Furthermore, we also overlaid on this plot six figures *G*_1_, *G*_2_, …, *G*_6_ (as defined in inset table in Figure 3) representing patterns of those probe sets that are unanimously declared as either rhythmic or non-rhythmic by all normalization methods within a given algorithm. For example, *G*_1_ (*Cyclic*) is a pattern of all probe sets that are declared as rhythmic by all normalization methods and all three algorithms. On the other hand, *G*_2_ (*Quasi Cyclic*) is a pattern of all probe sets that are declared as rhythmic by all normalization methods using ORIOS but not rhythmic under all normalizations methods when using JTK or RAIN. Since genes declared as rhythmic by JTK algorithm are also declared as rhythmic by RAIN algorithm, therefore we are describing only six patterns *G*_1_, *G*_2_, …, *G*_6_ and not eight patterns as one might expect.

In Figure 3, we interpret horizontal axis (Dim1) as the axis describing rhythmicity because all *a*_*n*_1 appear on the right hand side and almost all *a*_*n*_0 appear on the left hand side of the graph. Using *G*_1_, *G*_2_, …, *G*_6_, we see that Dim1 separates rhythmicity (Cyclic-shaped patterns) against non-rhythmicity (Flat-shaped patterns). Furthermore, it is interesting to note that rhythmic-shaped patterns (Cyclic, Quasi Cyclic, and Asymmetric) identified by ORIOS are located in the upper portion of the first quadrant of the MCA plot and the third quadrant exclusively consists of non-rhythmic patterns identified by ORIOS. Thus the first and the third quadrants of MCA plot appear to distinguish ORIOS from the others. The vertical axis (Dim2) may be interpreted as the axis drawing distinctions between ORIOS and RAIN algorithms. Lastly, it is clear from the MCA plot that ORIOS normalization methods are less separated than JTK or RAIN, i.e., rhythmic (and non-rhythmic) groups are more compact when using ORIOS, which is one more reason, in addition to the results provided in Larriba et al. (2016), to prefer ORIOS as the algorithm for detecting rhythmic genes.

To show that our proposed rhythmicity measure **M**_Robust_(*n, a*) is generally robust to the normalization methods, we computed the Spearman and Pearson correlation coefficients between **M**_Robust_(*n*_*i*_, *a*) and **M**_Robust_(*n*_*j*_, *a*), for all pairs of normalization methods *n*_*i*_, *n*_*j*_, *i* ≠ *j* and compared the correlations with those corresponding to the standard measure **M**(*n, a*). In addition to Spearman and Pearson correlation coefficient, we also computed the percent of concordance of rhythmic and non-rhythmic genes across all normalization methods using standard measure **M**(*n, a*) and the proposed robust measure **M**_*Robust*_(*n, a*). Due to space reasons, in the main paper we only present the results for ORIOS, i.e., *a* = *ORIOS*, but the results corresponding to JTK and RAIN are provided in the supporting materials.

**Figure 4:**
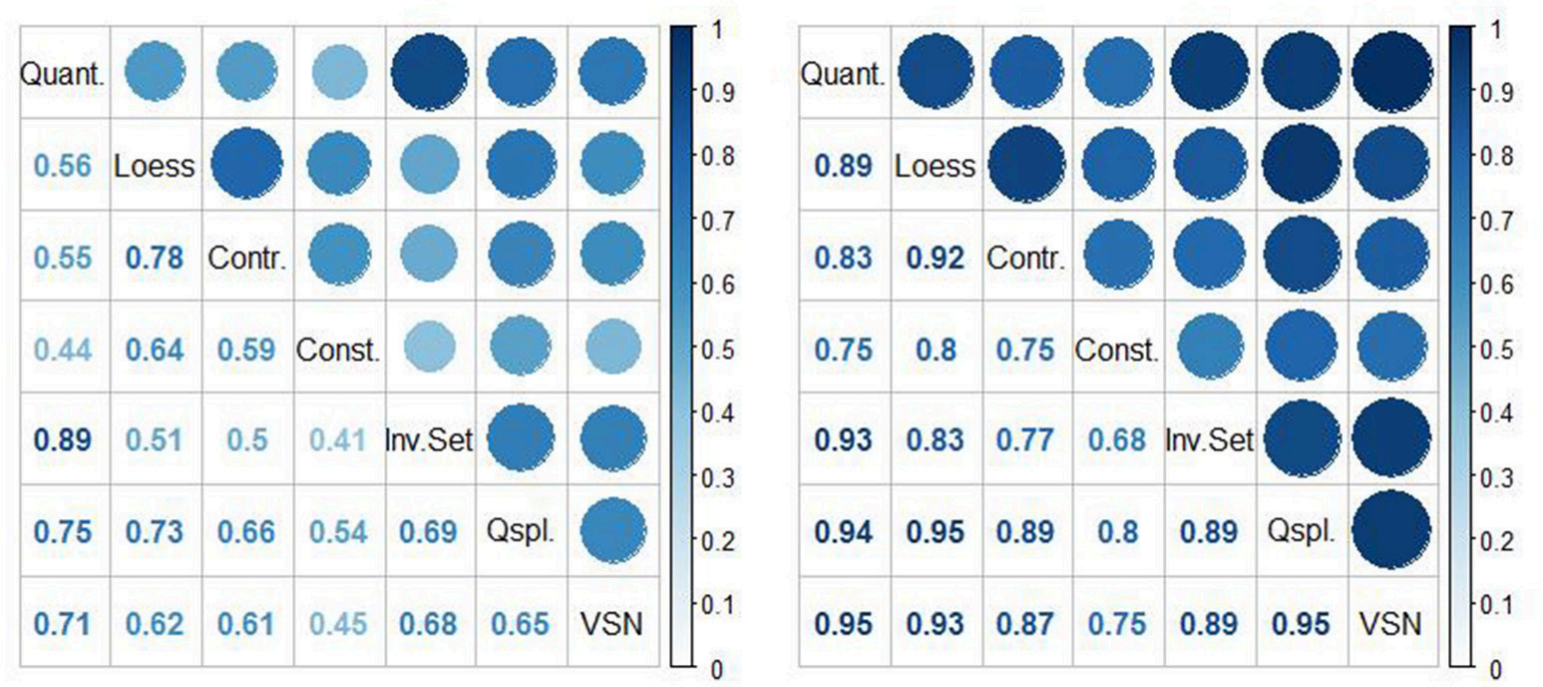
Spearman rank correlation coefficients between all pairs of normalization procedures considering the standard measure of rhythmicity ***M*** **(left)** and the proposed robust measure ***M***_***Robust***_ **(right)** for the ORIOS algorithm using the 15,369 probe sets, showing a highly increased consistency due to bootstrapping.

**Figure 5:**
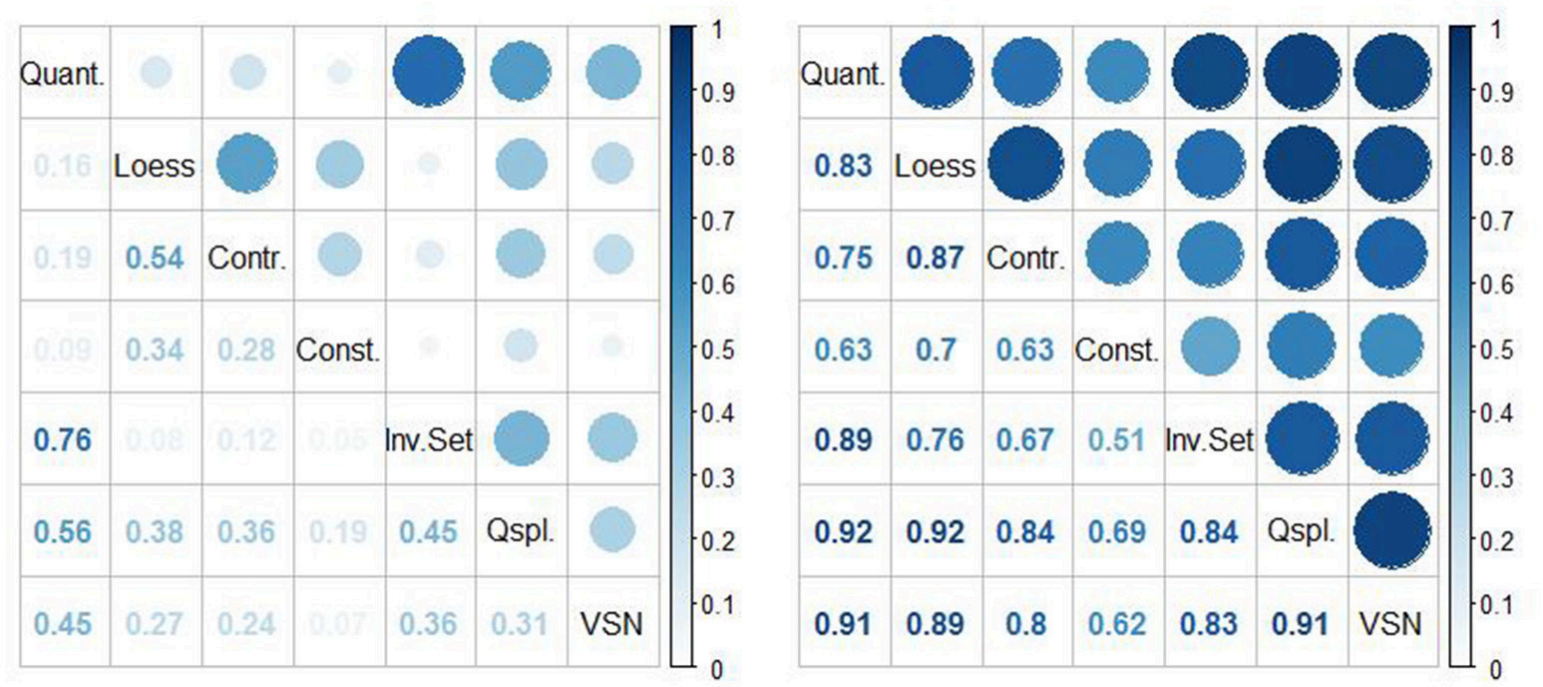
Pearson correlation coefficients between all pairs of normalization procedures considering the standard measure of rhythmicity ***M*** **(left)** and the proposed robust measure ***M***_***Robust***_ **(right)** for the ORIOS algorithm using the 15,369 probe sets. The robust measure shows a highly increased consistency among normalizations.

**Figure 6:**
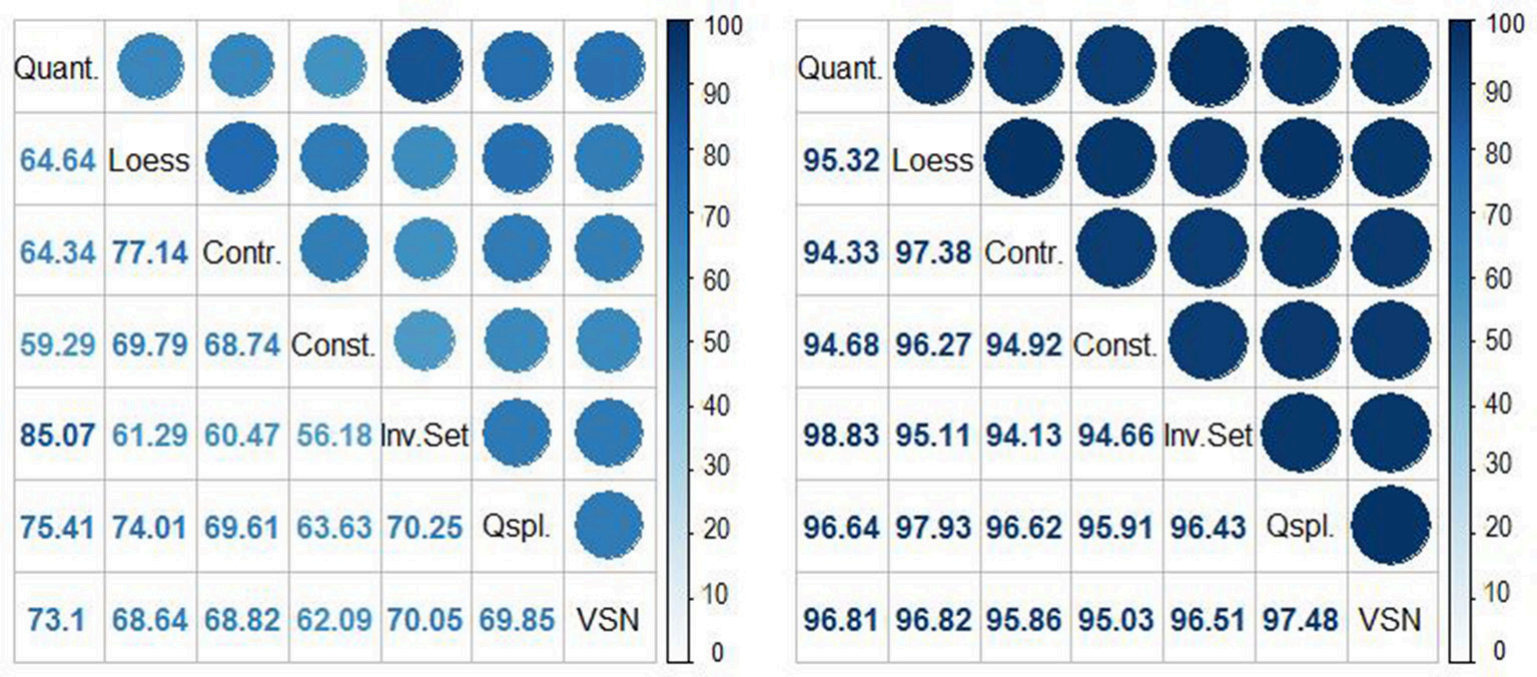
Percentage of (rhythmic and non-rhythmic) concordant probe sets before **(left)** and after **(right)** bootstrapping for all pairs of normalization procedures using the 15,369 probe sets. Bootstrapping increases significantly the concordance.

**Figure 7:**
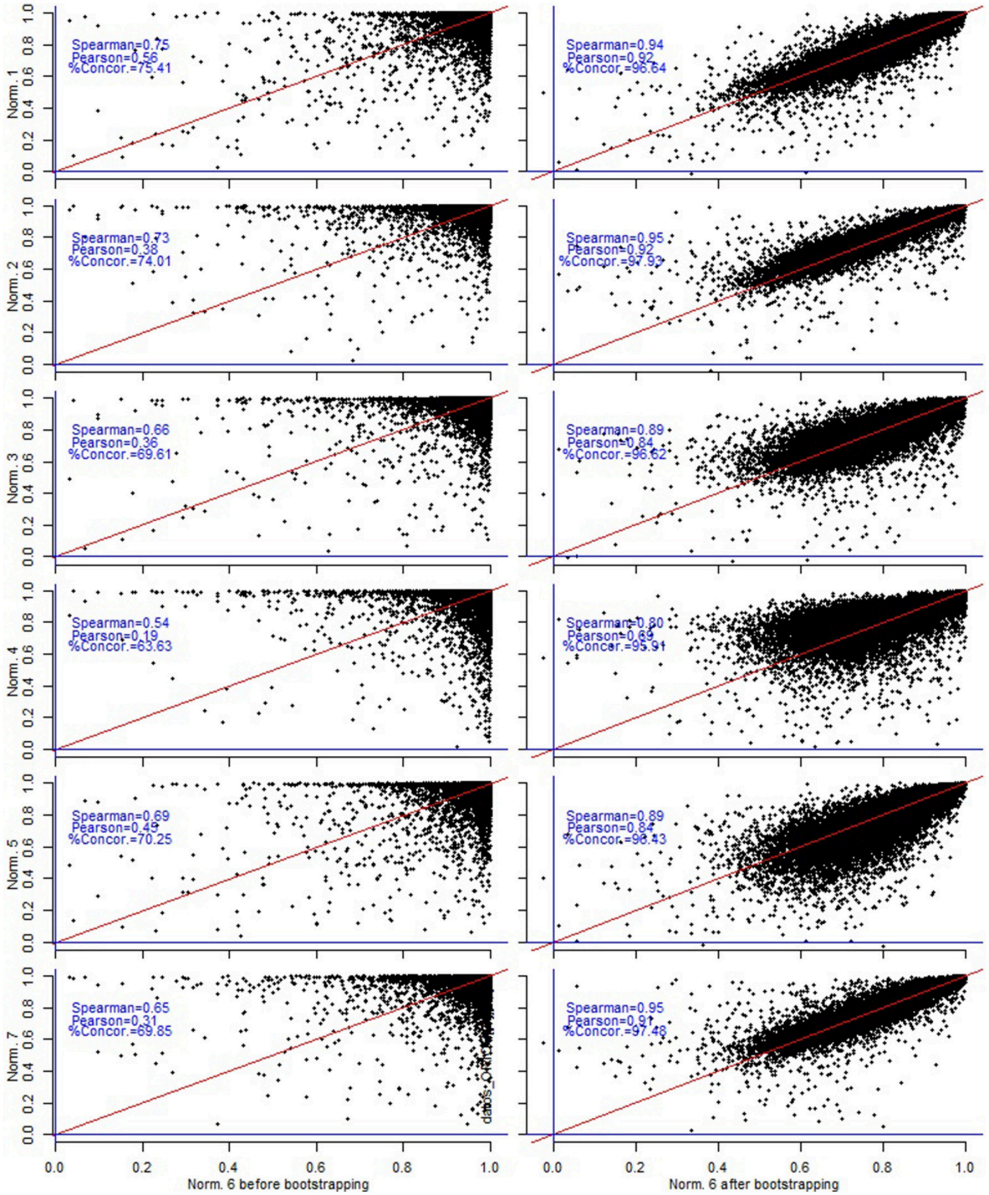
For each normalization method *n*, the **left** panels represent the pairwise scatter plots of [*M*^*g*^(*n, ORIOS*), *M*^*g*^(*Qspline, ORIOS*)] and the **right** panels represent the pairwise scatter plots of $\left[M_{Robust}^{g}\left(n,ORIOS\right),M_{Robust}^{g}\left(Qspline,ORIOS\right)\right].$ Red line is the 45° diagonal and the blue lines are the Cartesian axes. Right side scatter plots show a much more elliptical shape and a higher correlation indicating higher consistency between *Qspline* and the other normalizations.

In our correlation and concordance analyses reported in Figures 4–**6**, we limited to only those probe sets that were considered to be rhythmic by the criterion *M*^*g*^(*n, ORIOS*) ≥ 0.99 for at least one normalization method *n*. Thus we limited to 15,369 probe sets out of 45,101. The left hand panels of Figures 4–**6** correspond to **M**(*n, ORIOS*), whereas the right hand panels correspond to **M**_*Robust*_(*n, ORIOS*). From these figures it is clear that both correlation and the concordance increase substantially for every pair of normalization methods from the left panel to the right panel. To illustrate this fact, observe that the Spearman correlation between **M**(*Qspline, ORIOS*) and **M**(*VSN, ORIOS*) is 0.65 (left panel of Figure 4). However, the Spearman correlation between **M**_*Robust*_(*Qspline, ORIOS*) and **M**_*Robust*_(*VSN, ORIOS*) is 0.95 (right panel of Figure 4), which is a substantial increase. The increase is even more dramatic if one were to consider the Pearson correlation coefficient which increases from 0.31 to 0.91 (Figure 5). Even the percentage of concordant genes between these normalization procedures increases dramatically by more than 27%, from 69.85 to 97.48% (Figure 6). For each normalization method *n*, these increases are further illustrated using scatter plots of the pairs [*M*^*g*^(*n, ORIOS*), *M*^*g*^(*Qspline, ORIOS*)] (left panel) and $\left[M_{Robust}^{g}\left(n,ORIOS\right),M_{Robust}^{g}\left(Qspline,ORIOS\right)\right]$ (right panel) in Figure 7. The scatter plots on the left generally display highly non-elliptic scatter of points with no clear correlation. However, the scatter plots on the right panel which correspond to our robust method, appear to be very elliptic and in some cases with very small minor axis. As a by-product, these scatter plots together with Figures 4–6, imply that among the seven normalization methods, the *Constant* and *Invariant Set* normalization methods may be the least preferred normalization methods as the robust measure corresponding to these methods seem to be least correlated with others. Similar dramatic increases are also seen for JTK and RAIN as described in the figures in the online Supplementary Material (Figures S2–S9).

For the dataset corresponding to the mouse pituitary data (see section 2.2 in the Supplementary Material) and the NIH3T3 mouse cell line data (section 2.3 in the Supplementary Material) we obtain similar increases. For example, for the mouse pituitary, the Spearman rank correlation between *Qspline* and *VSN* (for the ORIOS algorithm) increases from 0.4 for the standard measure to 0.87 for the robust measure, while the Pearson coefficient increases from 0.21 to 0.82 and the concordance percentage goes from 62.56 to 98.67%. For the NIH3T3 cell lines, the same measures increase from 0.38 to 0.82 for the Spearman rank correlation, from 0.23 to 0.75 for the Pearson correlation and from 62.01 to 99.06% for the concordance percentage.

## 4. Discussion

Determination of circadian clock genes is an important problem in various fields, especially clinical pharmacology (Zhang et al., 2014; Chen and Yang, 2015) where they play an important role in drug delivery and medicine. However, identification of such rhythmic genes in genome-wide studies involving oscillatory systems has been a long standing problem. While it is well-acknowledged in the literature that normalization methods play an important role in determining differentially expressed genes in a pair of conditions (Cheng et al., 2016), as demonstrated in this paper, they play a bigger role in determining rhythmic genes in long-series time-course experiments. For example, as observed in Figure 1 and as seen from Spearman and Pearson correlations reported in Figures 4, 5, the rhythmicity of a gene can be dramatically affected by the normalization method used. This is the first paper we know that studies this problem for long-series time-course experiments and provides a simple bootstrap based methodology that correlates well across various normalization methods. The pairwise correlations among the normalization methods improve dramatically by using our proposed methodology. For example, the Pearson correlation coefficient between *Qspline* and *VSN* nearly triples from 0.31 to 0.91 after applying our robust methodology. All statistical decision rules require a user-supplied threshold when making inferences and the proposed methodology is no exception. The threshold of 0.99 used in our criterion for rhythmicity corresponds to 1% level of significance and is largely motivated by the specificity and sensitivity findings of Larriba et al. (2016).

Since the Spearman correlation coefficient is based on the ranks, we therefore make a crucial observation from Figure 4 (and Figures S10, S22 in the Supplementary Material) that rank of rhythmicity of a gene is correlated across all normalization methods considered here when our bootstrap based methodology is applied. Thus, if a gene has a high rank of rhythmicity under one normalization method, then it is also expected to have a similarly high rank of rhythmicity under other normalization methods. Conversely, if a gene has a very low rhythmicity rank under one normalization method then it will likely to have low rank under a different normalization method. To illustrate this point, consider the two genes described in the motivating figure of this paper (Figure 1). As noted earlier, under the standard criterion *M*^*g*^(*n, ORIOS*) ≥ 0.99, the rhythmicity calls on these two genes highly depended upon the normalization method *n*. However, under the criterion $M_{Robust}^{g}\left(n,ORIOS\right)\geq 0.99$, neither of these genes are considered to be rhythmic. Specifically, using the normalization methods used earlier for Figure 1, we obtained the following robust rhythmicity measures $M_{Robust}^{Serpina3k}\left(Quantile,ORIOS\right)=\;0.127,\;M_{Robust}^{Serpina3k}\left(Constant,ORIOS\right)=\;0.367$, $M_{Robust}^{Maml1}\left(Loess,ORIOS\right)=0.675$, and $M_{Robust}^{Maml1}\left(InvariantSet,ORIOS\right)=0.614$. None of these numbers exceed 0.99.

Observe that, unlike Figures S8, S9 in the Supplementary Material for JTK and RAIN algorithms, none of the scatter points in the right panel of Figure 7 for ORIOS take negative values, except for one, thus indicating that ***M***_***Robust***_(*n, ORIOS*) almost always takes positive values for all normalization methods *n*. However, ***M***_***Robust***_(*n, JTK*) and ***M***_***Robust***_(*n, RAIN*) take negative values. Notice that something similar happens for both the mouse pituitary (Figures S19–S21 in the Supplementary Material) and NIH3T3 cell line datasets (Figures S31–S33). Since ${\text{M}}_{\text{Robust}}\left(n,a\right)=\hat{𝔼}\left(\hat{\theta }\left(n,a\right)\right)-\hat{RMSE}\left(\hat{\theta }\left(n,a\right)\right),$ therefore the variability in *p*-values for tests for rhythmicity using JTK and RAIN methods is larger than the corresponding estimated *p*-values. Thus the JTK and RAIN methods produce *p*-values that are subject to higher variation and uncertainty than the expected *p*-values. This is in sharp contrast to ORIOS which almost always produced *p*-values subject to smaller variability than the expected *p*-values. This is one more reason, in addition to the results provided in Larriba et al. (2016), to prefer ORIOS as the method for detecting rhythmic genes.

The bootstrap methodology introduced in this paper is computationally efficient. For each of the datasets analyzed in this paper, the method required ~70 min of CPU time to generate and process 45,101 probe sets on Windows 7 Professional 3.60 GHz dual processors computer with disk space using 100 bootstrap samples.

From our investigation of real data and the bootstrap simulated data, we find that our bootstrap procedure provides a simple and a convenient way to simulate oscillatory signals that potentially resemble realistic patterns of expression. Thus, as a secondary contribution, in this paper we introduced a bootstrap methodology that not only provides methodology to detect rhythmic genes but it also allows researchers to conduct simulation studies to generate realistic rhythmic patterns. Notice also that, although for illustration and clarity purposes, in this paper we focused on gene expression studies (such as microarray and RNA-seq), the methodology described here is applicable to any modern high-throughput technology involving oscillatory systems. For example, it can potentially be used for analyzing continuous time microbiome data, such as those obtained in Caporaso et al. (2011).

## Author contributions

CR, MF, and SP: Conceived aims, conceptual design, data analysis, interpretation of the results, wrote and approved manuscript; YL: Processed original data, generated simulations, analyzed data, interpreted results, wrote and approved manuscript.

### Conflict of interest statement

The authors declare that the research was conducted in the absence of any commercial or financial relationships that could be construed as a potential conflict of interest.
