# Supplementary material for: A Bootstrap Based Measure Robust to the Choice of Normalization Methods for Detecting Rhythmic Features in High Dimensional Data
Source: Front Genet. 2018 Feb 2;9:24. doi: 10.3389/fgene.2018.00024 (PMC5801422; doi:10.3389/fgene.2018.00024)
Supplement: Supplementary file 1 [file Presentation1.pdf]

Supporting material for “A Bootstrap Based Measure Robust to the Choice of Normalization Methods for Detecting Rhythmic Features in High Dimensional Data”

This supporting material text is divided in two sections. In Section 1 we detail the work-flow to pre-process microarrays including additional figures referenced in the main article. In Section 2 we describe additional numerical results for the mouse liver dataset obtained from JTK and RAIN across the seven normalization methods. Additionally, this section includes numerical results for ORIOS, JTK and RAIN across the seven normalization methods for two additional microarray datasets derived from mouse pituitary tissue and from NIH3T3 mouse cell lines considered in Hughes *et al.* (2009) to reinforce the performance of the bootstrap based methodology proposed in this paper.

1 WORK-FLOW OF THE PRE-PROCESSING PROCEDURE

Figure S1 illustrates the work-flow of the pre-processing procedure, which includes background correction, normalization and summarization steps, and from which arises the bootstrap based methodology described in the main document. More specifically, let **R** denote the tri-dimensional array of raw intensities obtained from a reference high-throughput microarray experiment. Data in **R** are expressed at probe level, i.e. gene-intensity values in **R** depends on *probe* and *array* effects. Specifically, R^g_{pt} denotes the raw intensity value for gene g on *probe* p at time point (*array*) t, where $g = 1, \ldots, G$, $p = 1, \ldots, P$ and $t = 1, \ldots, T$. Thus, **R** is the input of the pre-processing procedure. Two subsequent tri-dimensional arrays of intensities, namely **X** and **Z**, are directly derived after background correction and normalization steps respectively, both expressed at probe level. The output of pre-processing is a matrix of gene-expression values expressed at gene (or probe set) level, denoted as **S**, which is obtained from **Z** using a median polish algorithm, (Emerson and Hoaglin, 1983).

In this work, the normalization methods are implemented together with the background correction and summarization steps along the lines proposed in Irizarry *et al.* (2003) and making use of the R-package *Affy* (Gautier *et al.*, 2004) from the Bioconductor Project (Ihaka and Gentleman, 1996).

2 ADDITIONAL METHODOLOGY RESULTS

Due to space limitations and the fact that robustness results were similar for the microarray datasets considered, the main document provided all our results for ORIOS algorithm in the mouse liver dataset only. In this supplementary text we provide analogous graphs when JTK and RAIN were used as the algorithms for identifying rhythmic patterns in the mouse liver tissue (Section 2.1) as well as the results obtained for ORIOS, JTK and RAIN in two additional microarray datasets from mouse pituitary tissue (Section 2.2) and mouse cell lines NIH3T3 (Section 2.3). These datasets have been analysed in literature to detect rhythmicity (Hughes *et al.*, 2009, 2010; Larriba *et al.*, 2016). Specifically, Larriba *et al.* (2016) claimed that mouse pituitary tissue and NIH3T3 cell lines presented a notably lower number of rhythmic genes than mouse liver did, and that gene-expressions from those datasets were also subject to higher noise levels.

2.1 Mouse liver tissue

Similar to the correlation and concordance analyses reported in Figures 4, 5 and 6, we limited to only those probe sets that were considered to be rhythmic by the criterion $M^g(n, ORIOS) \geq 0.99$ for at least one normalization method n. Thus, we limited to 15369 probe sets out of 45101. Similarly, when considering JTK (RAIN) method, we limited to only those probe sets that were considered to be to be rhythmic by the criterion $M^g(n, JTK(RAIN)) \geq 0.99$ for at least one

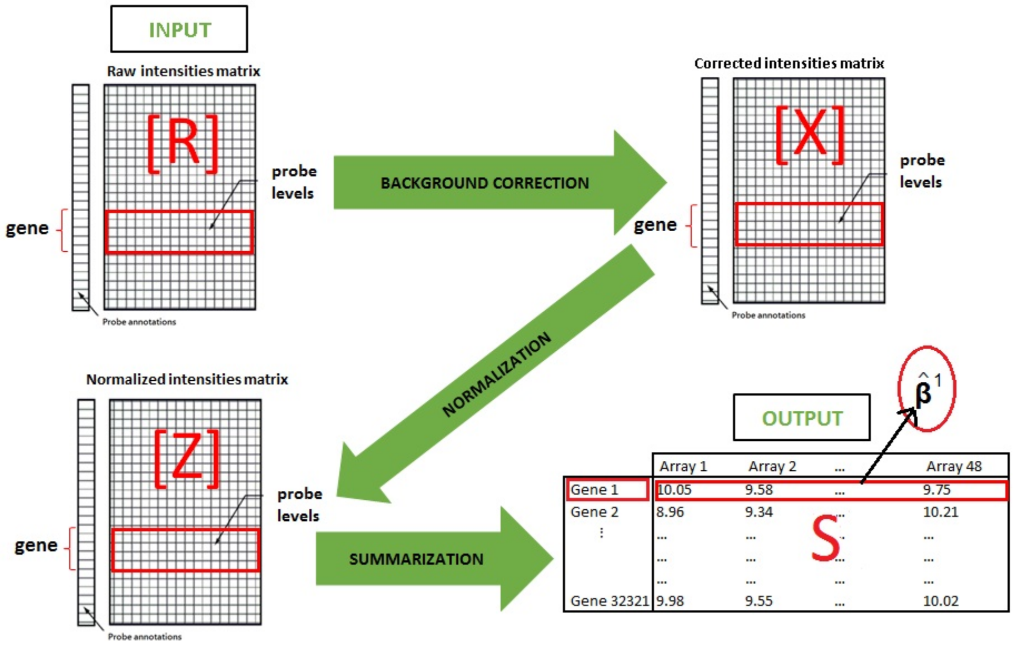

Figure S1: Work-flow of pre-processing procedure from raw intensities **R** to gene-expression matrix **S**

normalization method n. Thus in the case of JTK we limited to 5875 probe sets, whereas in the case of RAIN we limited to 16272 probe sets.

Analogous to Figures 4, 5 and 6 in the main paper, we obtained Figures S2, S3 and S4 for JTK and Figures S5, S6 and S7 for RAIN.

Consistent with the results observed for ORIOS, the Spearman and the Pearson correlation coefficients as well as the percentage of concordant probe sets increase substantially from the left panel to the right for JTK as well as RAIN algorithms. Furthermore, similar to the scatter plots seen in Figure 7 for ORIOS in the main paper, we obtain Figures S8 and S9 for JTK and RAIN respectively, the scatter plots on the right panel are more elliptic than the ones on the left panel for both JTK and RAIN. However, unlike the right panel of Figure 7 for ORIOS, the right panels of Figures S8 and S9 for JTK and RAIN take negative values, i.e. $\mathbf{M}_{\text{Robust}}(n, JTK(RAIN)) \leq 0$. Since

$$\mathbf{M}_{\text{Robust}}(n,a) = \widehat{\mathbb{E}}(\hat{\boldsymbol{\theta}}(n,a)) - \widehat{RMSE}(\hat{\boldsymbol{\theta}}(n,a)), \tag{S1}$$

therefore the variability in p-values for tests for rhythmicity using JTK and RAIN methods is larger than the estimated p-values using these two methods. Thus the JTK and RAIN methods produce p-values that are subject to higher variation than the expected p-values. This is in sharp contrast to ORIOS which always produced p-values subject to smaller variability than the expected p-values. Thus ORIOS always produces p-values with higher signal to noise ratio than JTK and RAIN. This is one more reason, in addition to the results provided in Larriba *et al.* (2016), to prefer ORIOS as the method for detecting rhythmic genes.

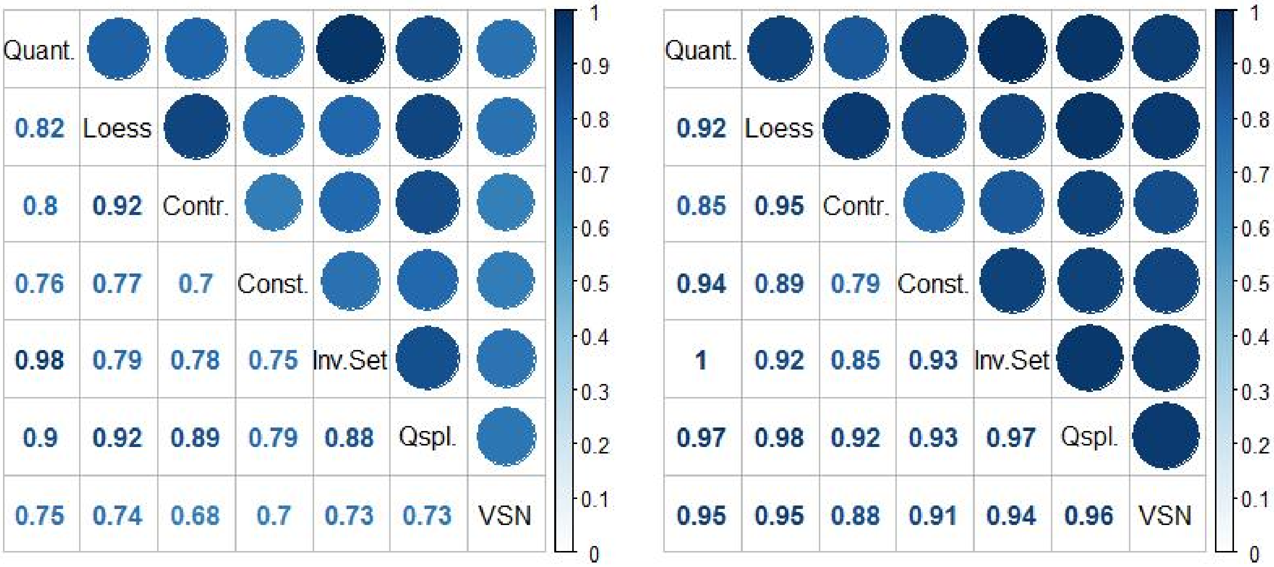

Figure S2: Spearman rank correlation coefficients between all pairs of normalization procedures considering the standard measure of rhythmicity (left) and the proposed robust measure (right) for the JTK algorithm, using the 5875 probe sets declared as rhythmic by JTK under at least one normalization procedure in the mouse liver dataset.

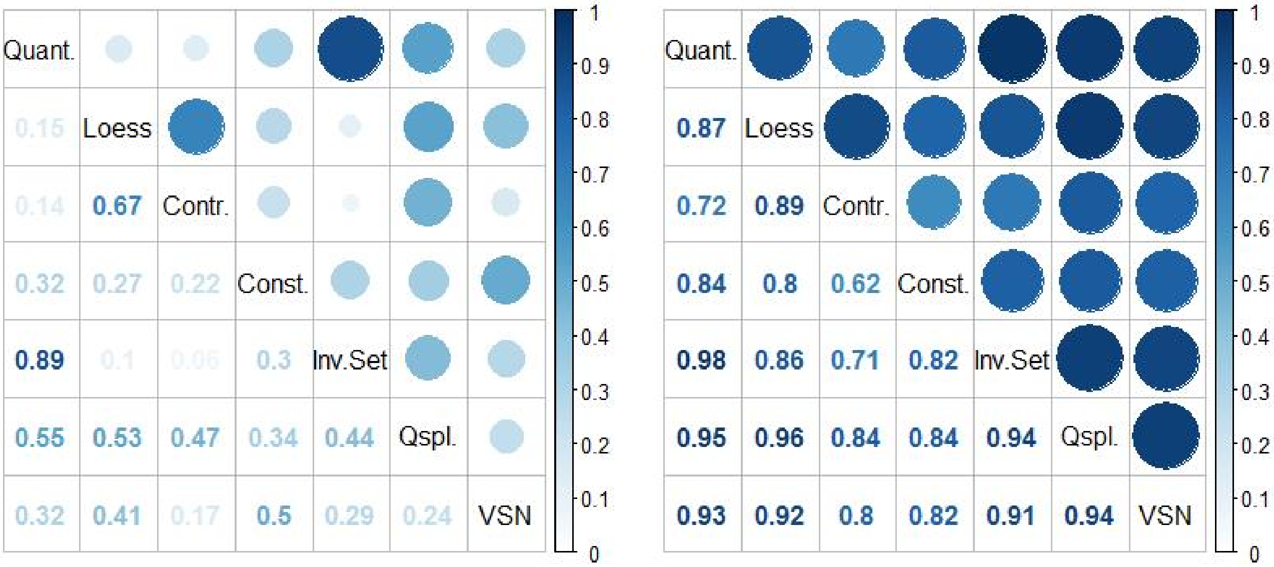

Figure S3: Pearson correlation coefficients between all pairs of normalization procedures considering the standard measure of rhythmicity (left) and the proposed robust measure (right) for the JTK algorithm, using the 5875 probe sets declared as rhythmic by JTK under at least one normalization procedure in the mouse liver dataset.

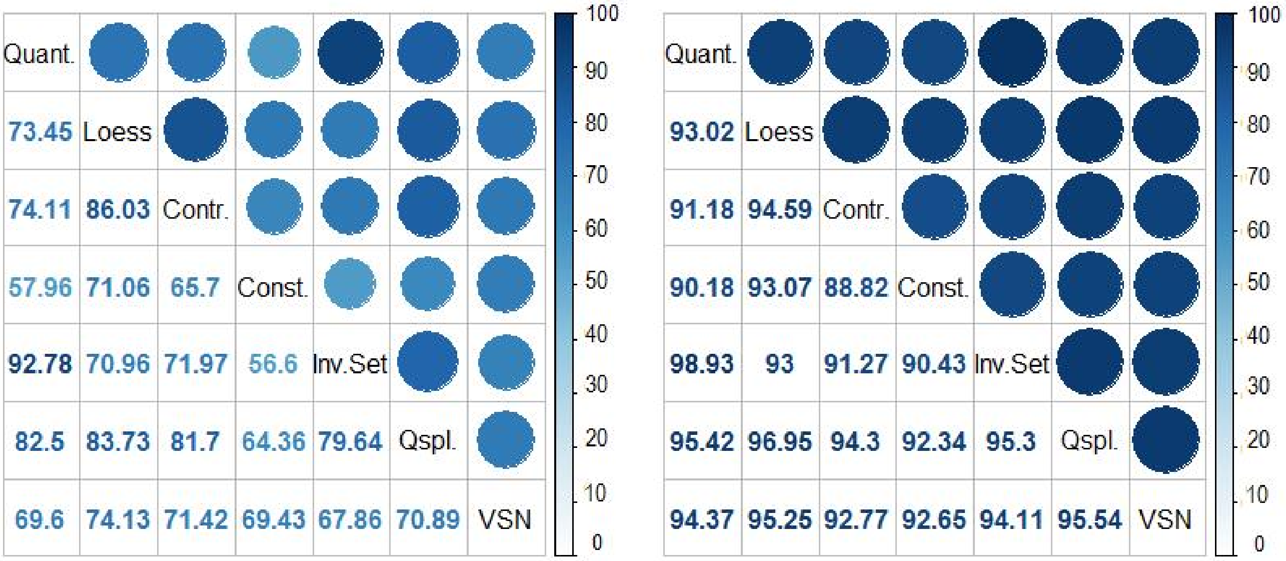

Figure S4: Percentage of (rhythmic and non-rhythmic) concordant probe sets before (left) and after (right) bootstrapping for all pairs of normalization procedures, using the 5875 probe sets declared as rhythmic by JTK under at least one normalization procedure in the mouse liver dataset.

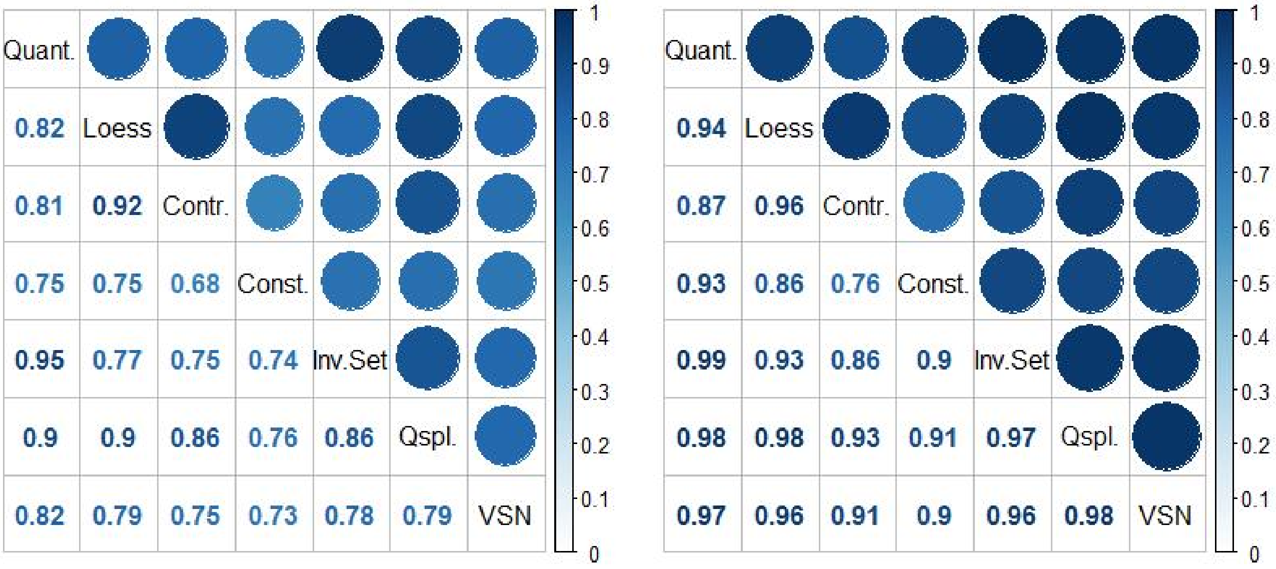

Figure S5: Spearman rank correlation coefficients between all pairs of normalization procedures considering the standard measure of rhythmicity (left) and the proposed robust measure (right) for the RAIN algorithm, using the 16272 probe sets declared as rhythmic by RAIN under at least one normalization procedure in the mouse liver dataset.

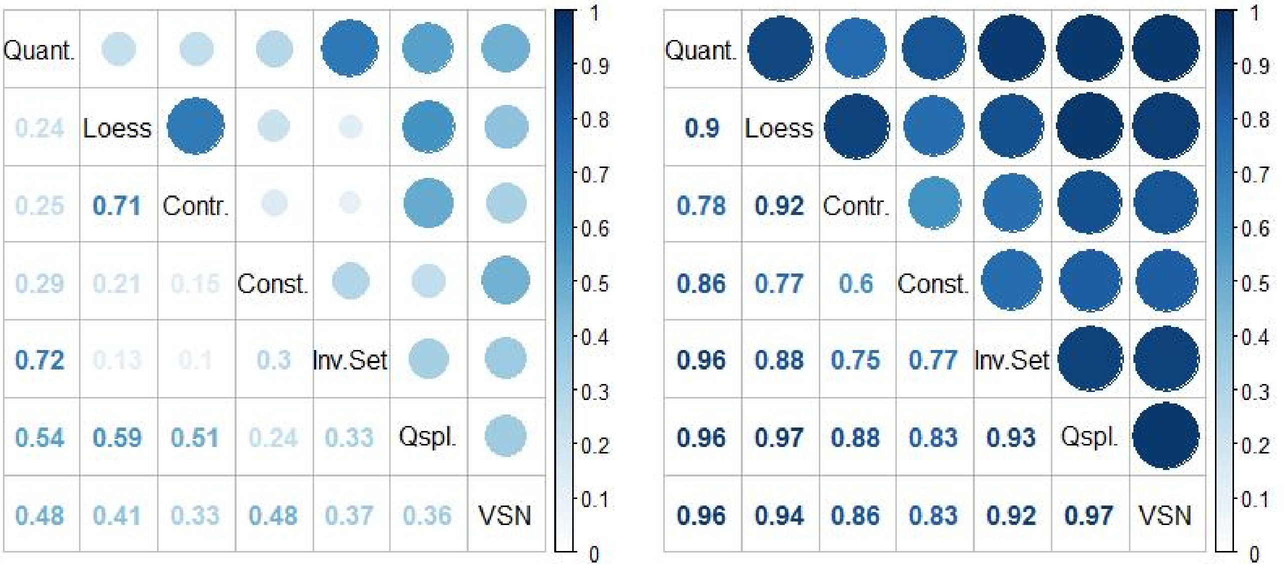

Figure S6: Pearson correlation coefficients between all pairs of normalization procedures considering the standard measure of rhythmicity (left) and the proposed robust measure (right) for the RAIN algorithm, using the 16272 probe sets declared as rhythmic by RAIN under at least one normalization procedure in the mouse liver dataset.

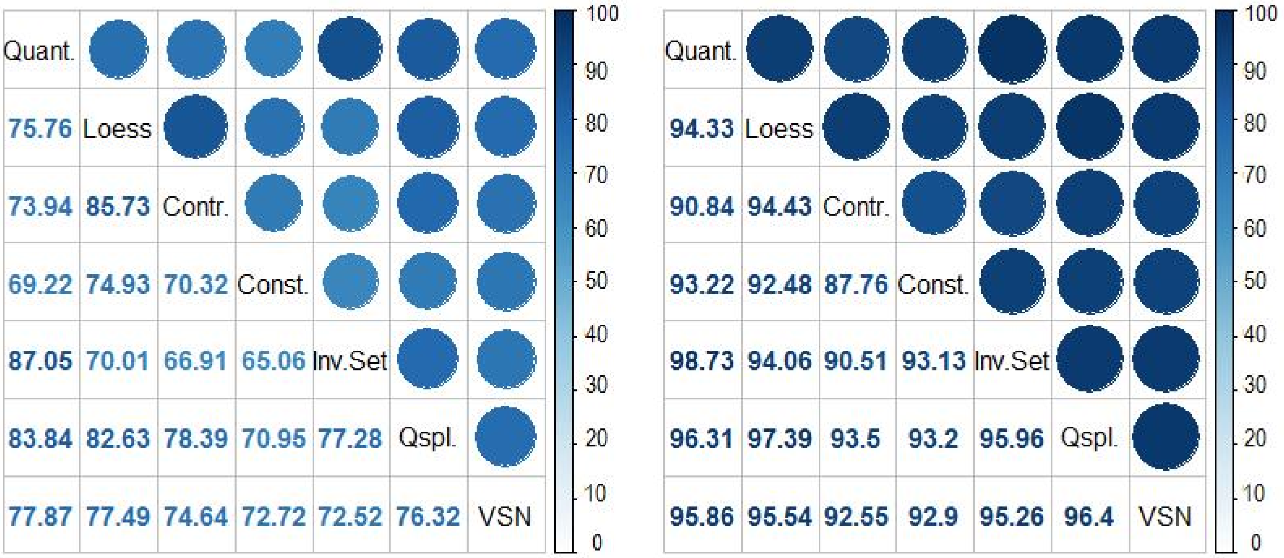

Figure S7: Percentage of (rhythmic and non-rhythmic) concordant probe sets before (left) and after (right) bootstrapping for all pairs of normalization procedures, using the 16272 probe sets declared as rhythmic by RAIN under at least one normalization procedure in the mouse liver dataset.

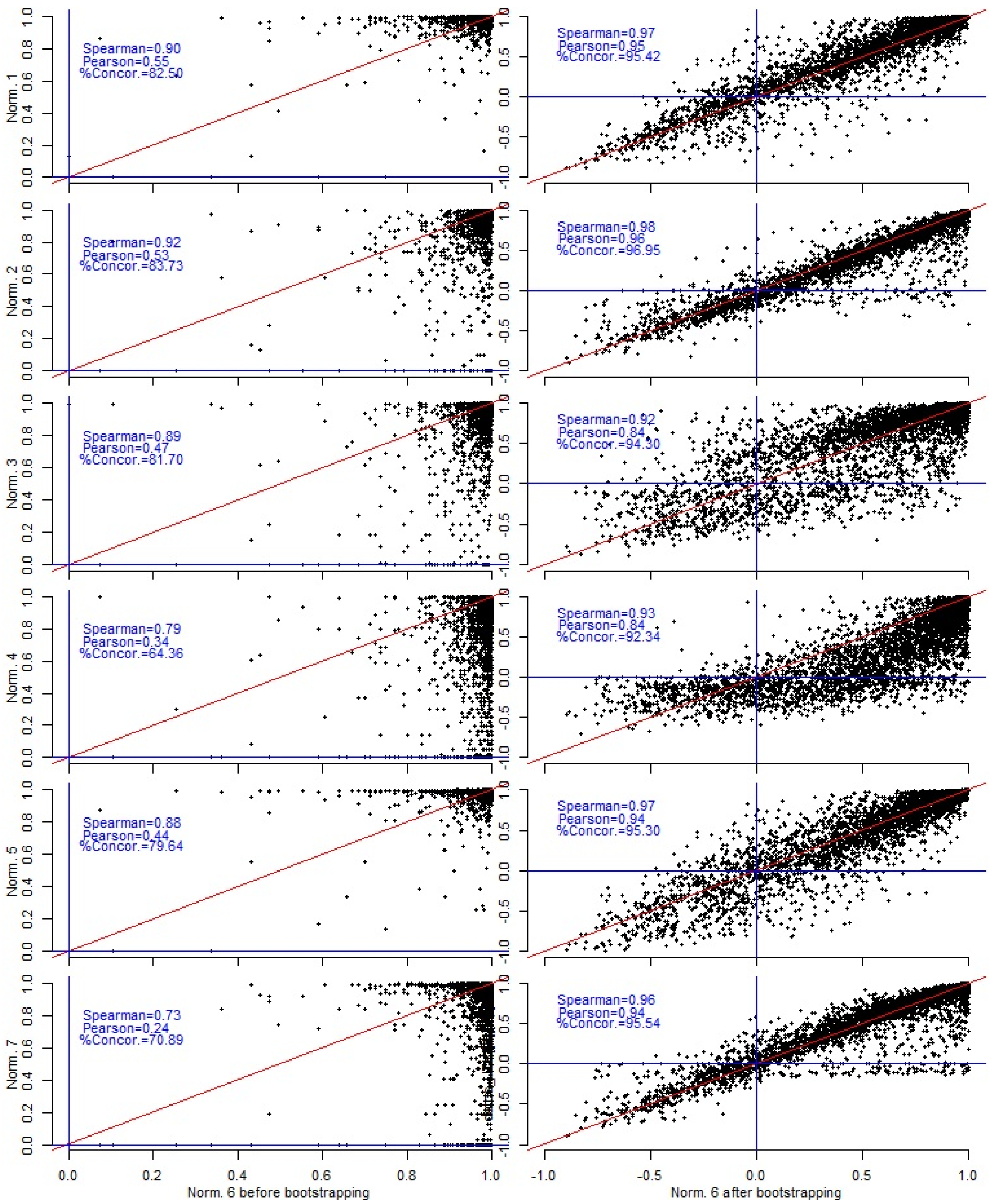

Figure S8: For each normalization method n, the left panels represent the pairwise scatter plots of $(M^g(n, JTK), M^g(Qspline, JTK))$ and the right panels represent the pairwise scatter plots of $(M^g_{Robust}(n, JTK), M^g_{Robust}(Qspline, JTK))$ for the mouse liver dataset. Red line is the 45° diagonal and the blue lines are the Cartesian axes.

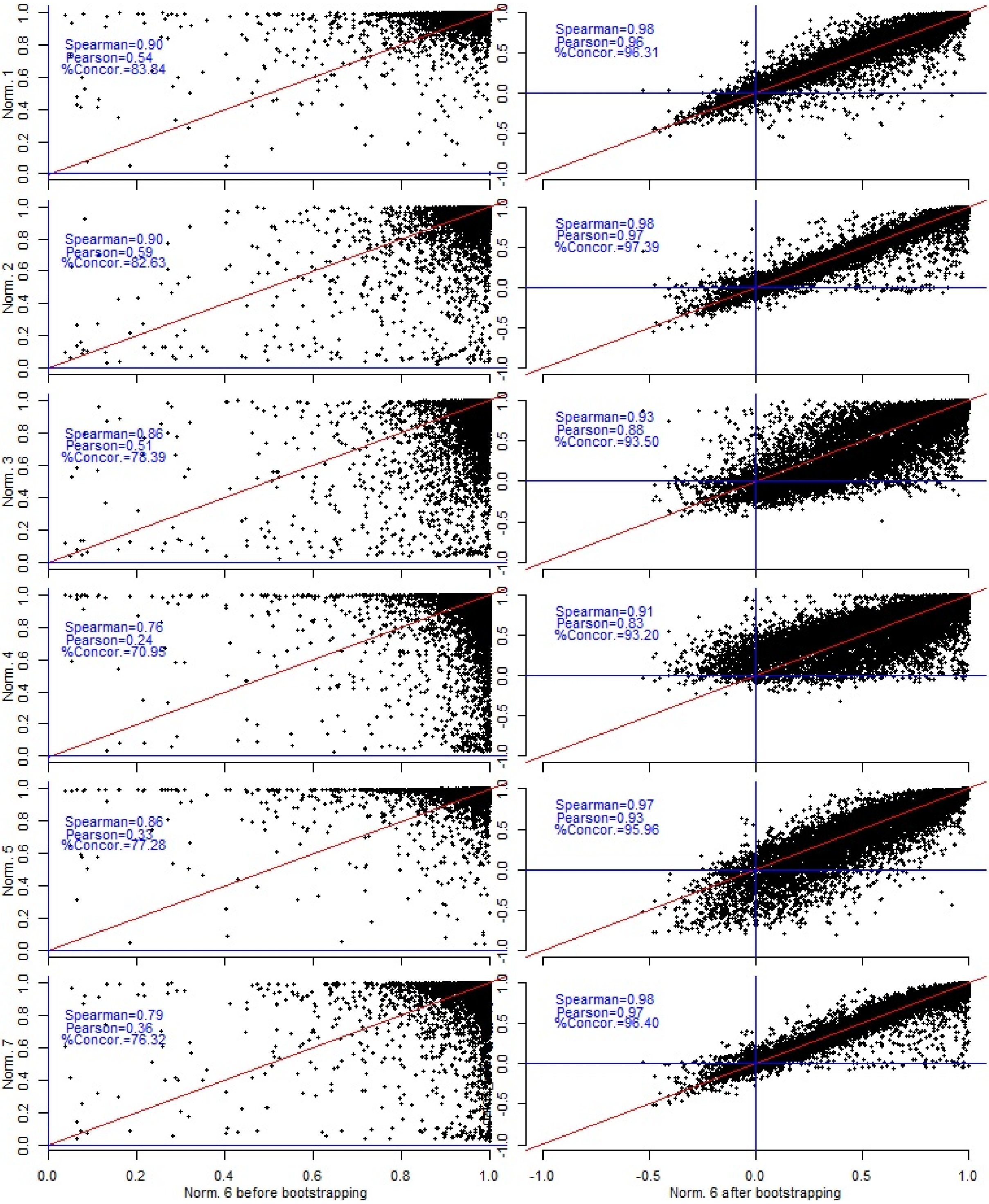

Figure S9: For each normalization method n, the left panels represent the pairwise scatter plots of $(M^g(n, RAIN), M^g(Qspline, RAIN))$ and the right panels represent the pairwise scatter plots of $(M^g_{Robust}(n, RAIN), M^g_{Robust}(Qspline, RAIN))$ for the mouse liver dataset. Red line is the 45° diagonal and the blue lines are the Cartesian axes.

2.2 Mouse pituitary tissue

Similar to the mouse liver data set, correlation and concordance analyses in mouse pituitary are limited for those probe sets that were considered to be rhythmic by the criterion $M^g(n, a) \geq 0.99$ for at least one of the normalization method n for each of the algorithms a. Thus in the case of ORIOS we limited to 7228 probe sets out of 45101, whereas in the case of JTK and RAIN we limited to 1081 and 10754 probe sets, respectively. Table S1 summarizes the results of three rhythmicity detection algorithms using the seven normalization methods in mouse pituitary.

Table S1. Number of genes in mouse pituitary originally detected as rhythmic by ORIOS, JTK and RAIN according to the different normalization strategies and $M^g(n, a) \geq 0.99$ for $g = 1, \ldots, 45101$

Normalization Strategy	ORIOS	JTK	RAIN
1 Quantile	3381	717	6571
2 Loess	3220	657	6185
3 Contrast	3484	650	6696
4 Constant	2844	447	5628
5 Invariant set	2608	643	5959
6 Qspline	3572	667	6271
7 VSN	2732	533	5354

Consistent with the results observed for the mouse liver data set, the Spearman and the Pearson correlation coefficients as well as the percentage of concordant probe sets increase notably from the left to the right panel regarding the algorithm considered, see Figures S10, S11 and S12 for ORIOS, Figures S13, S14 and S15 for JTK and Figures S16, S17 and S18 for RAIN. Furthermore, similar to the scatter plots seen in mouse liver (see Figures 7 -main paper-, S8 and S9), we obtain analogous scatter plots on which their right panels are more elliptic than the ones on the left panel, see Figures S19, S20 and S21 for ORIOS, JTK and RAIN results respectively. Analogous to mouse liver, JTK and RAIN methods produce p-values that are subject to higher variation and uncertainty than the expected p-values and that is in contrast to ORIOS which almost always produces p-values subject to smaller variability than the expected p-values.

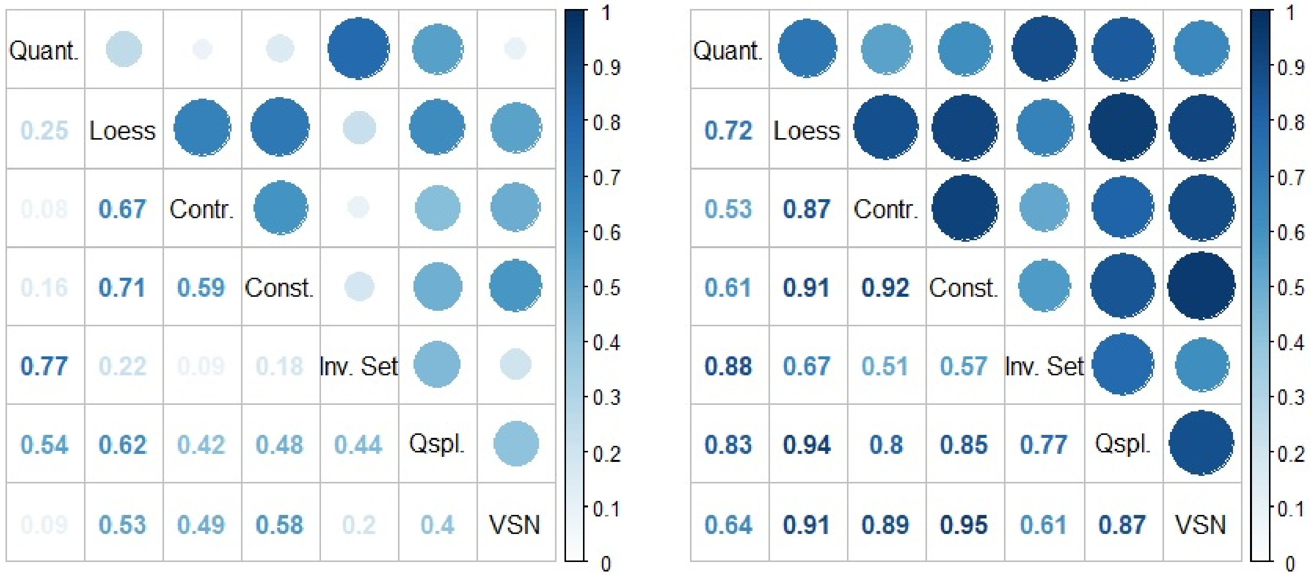

Figure S10: Spearman rank correlation coefficients between all pairs of normalization procedures considering the standard measure of rhythmicity (left) and the proposed robust measure (right) for the ORIOS algorithm, using the 7228 probe sets declared as rhythmic by ORIOS under at least one normalization procedure in the mouse pituitary dataset.

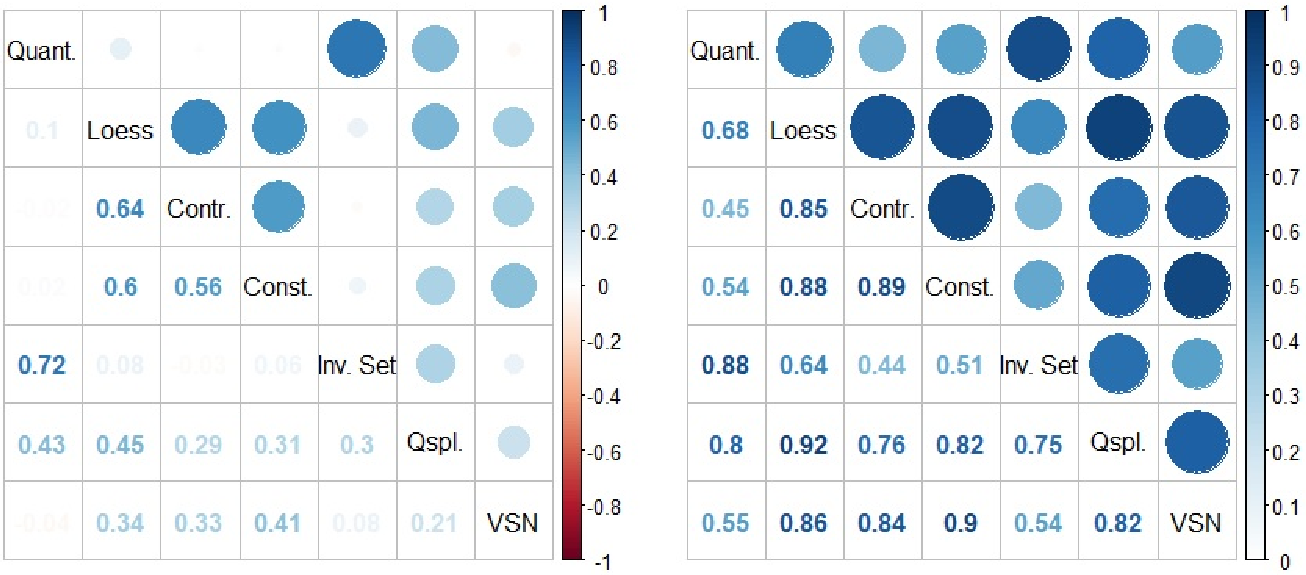

Figure S11: Pearson correlation coefficients between all pairs of normalization procedures considering the standard measure of rhythmicity (left) and the proposed robust measure (right) for the ORIOS algorithm, using the 7228 probe sets declared as rhythmic by ORIOS under at least one normalization procedure in the mouse pituitary dataset.

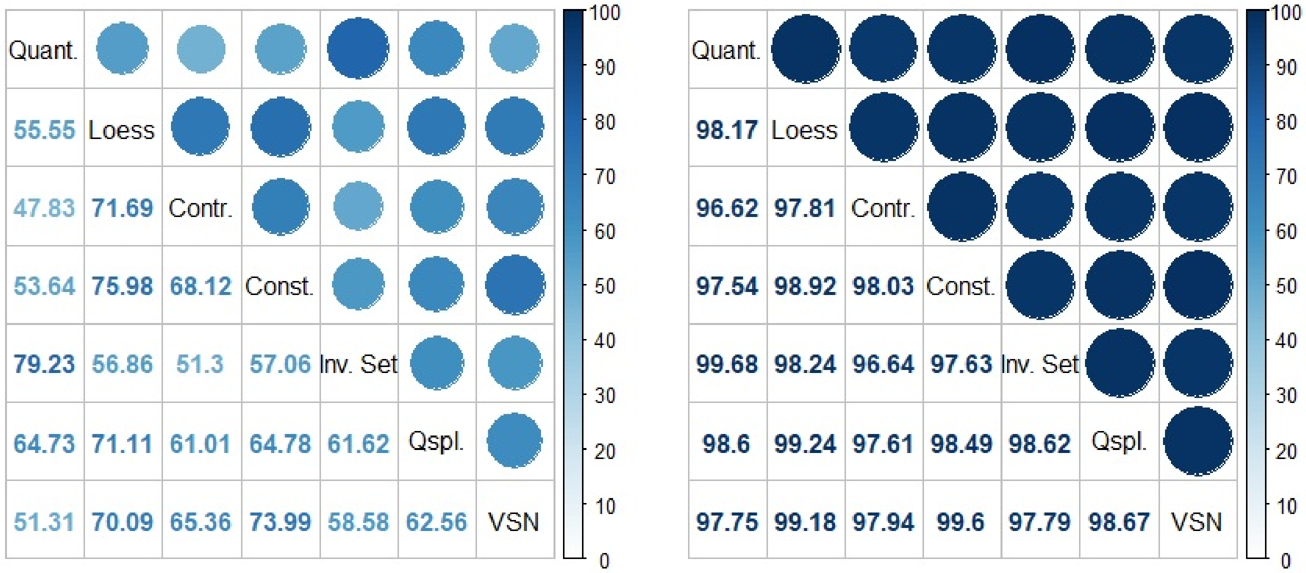

Figure S12: Percentage of (rhythmic and non-rhythmic) concordant probe sets before (left) and after (right) bootstrapping for all pairs of normalization procedures using the 7228 probe sets declared as rhythmic by ORIOS under at least one normalization procedure in the mouse pituitary dataset.

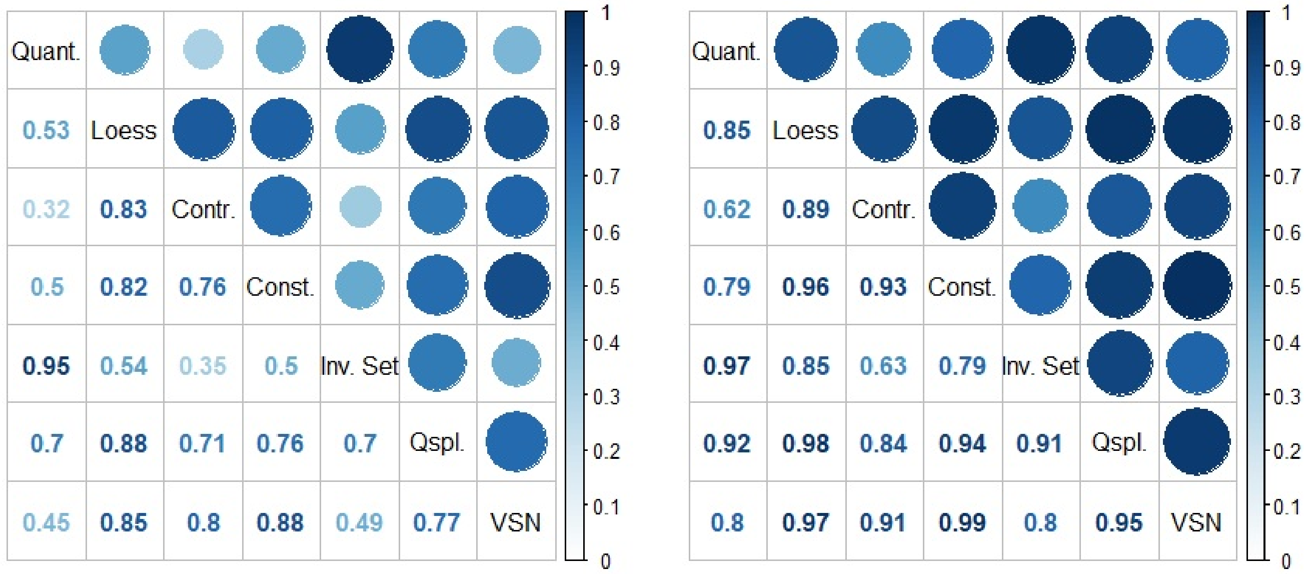

Figure S13: Spearman rank correlation coefficients between all pairs of normalization procedures considering the standard measure of rhythmicity (left) and the proposed robust measure (right) for the JTK algorithm, using the 1081 probe sets declared as rhythmic by JTK under at least one normalization procedure in the mouse pituitary dataset.

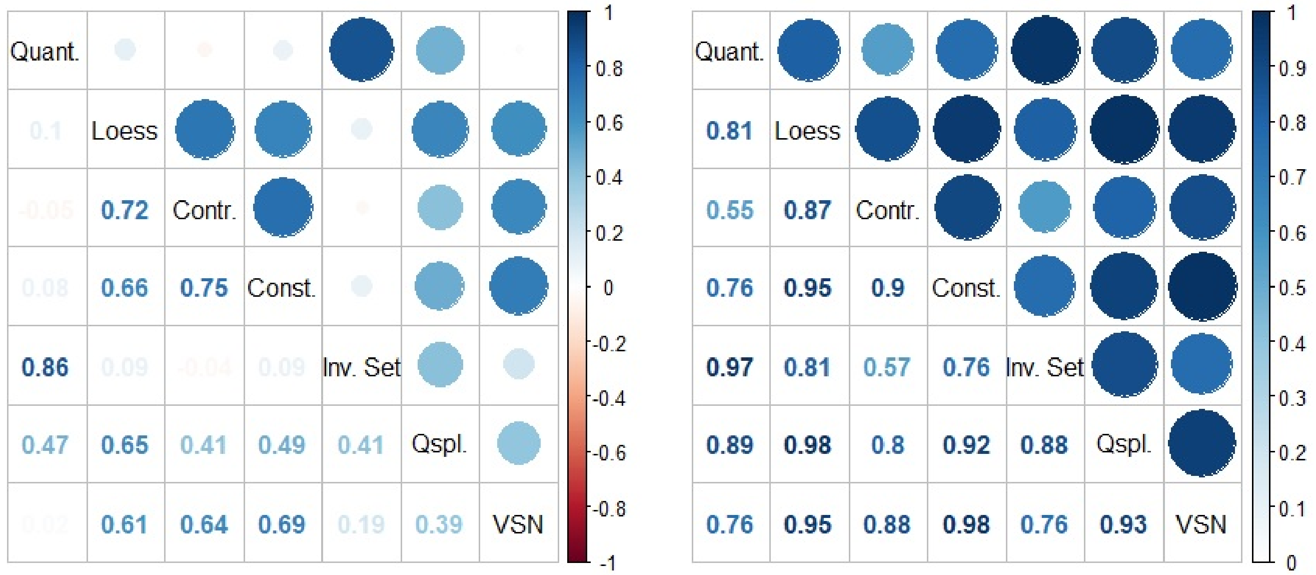

Figure S14: Pearson correlation coefficients between all pairs of normalization procedures considering the standard measure of rhythmicity (left) and the proposed robust measure (right) for the JTK algorithm, using the 1081 probe sets declared as rhythmic by JTK under at least one normalization procedure in the mouse pituitary dataset.

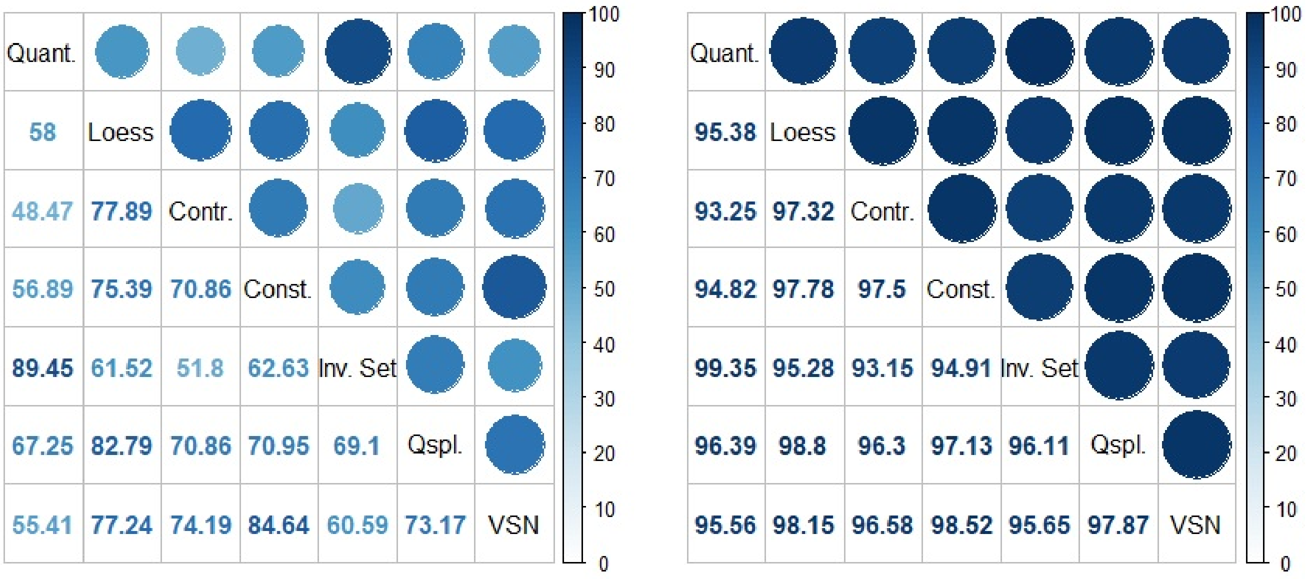

Figure S15: Percentage of (rhythmic and non-rhythmic) concordant probe sets before (left) and after (right) bootstrapping for all pairs of normalization procedures, using the 1081 probe sets declared as rhythmic by JTK under at least one normalization procedure in the mouse pituitary dataset.

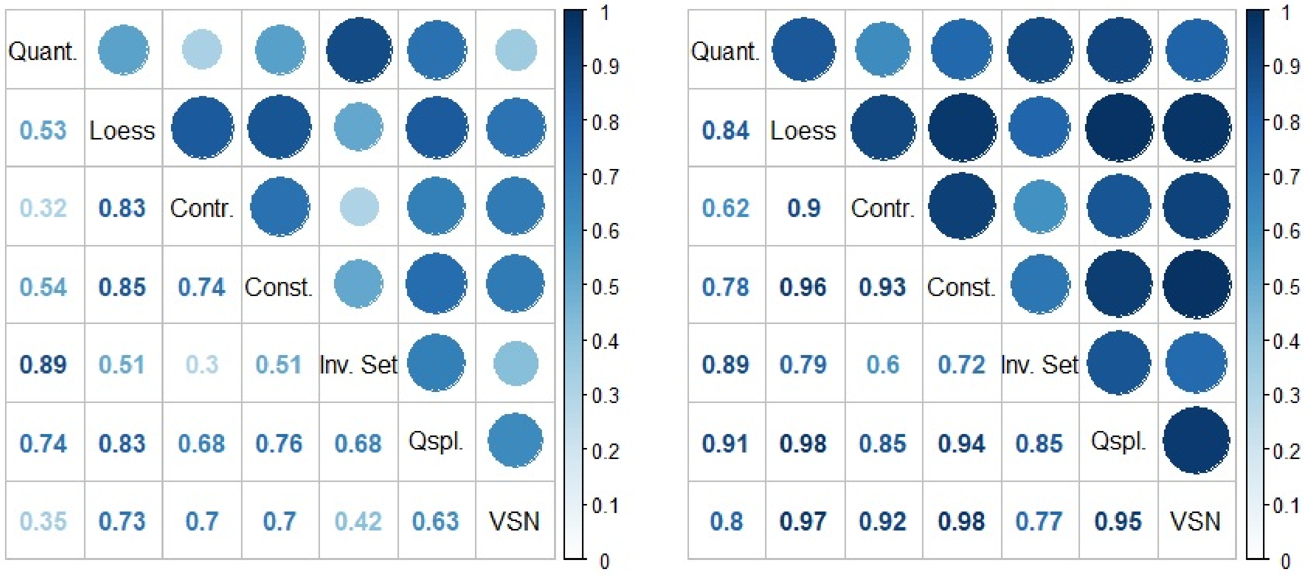

Figure S16: Spearman rank correlation coefficients between all pairs of normalization procedures considering the standard measure of rhythmicity (left) and the proposed robust measure (right) for the RAIN algorithm, using the 10754 probe sets declared as rhythmic by RAIN under at least one normalization procedure in the mouse pituitary dataset.

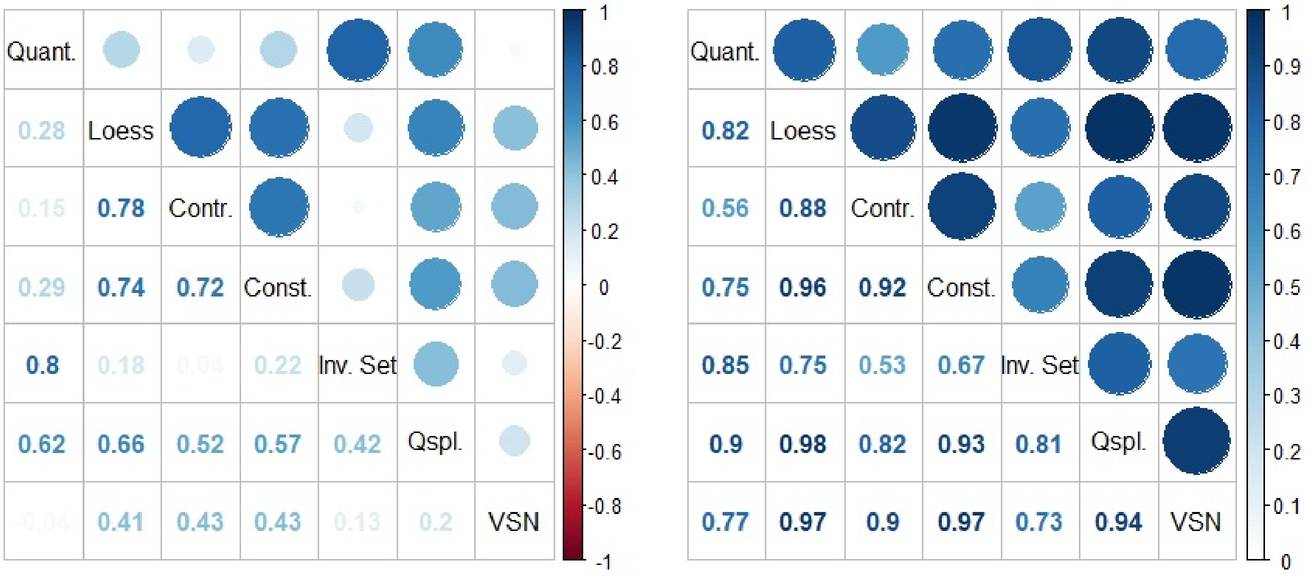

Figure S17: Pearson correlation coefficients between all pairs of normalization procedures considering the standard measure of rhythmicity (left) and the proposed robust measure (right) for the RAIN algorithm, using the 10754 probe sets declared as rhythmic by RAIN under at least one normalization procedure in the mouse pituitary dataset.

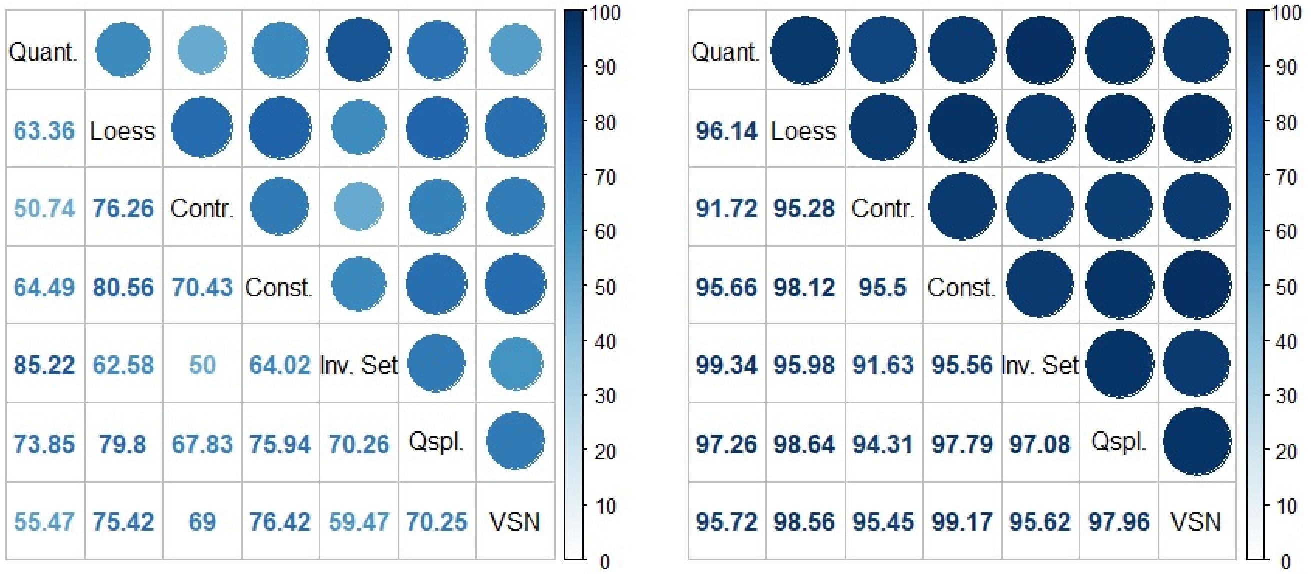

Figure S18: Percentage of (rhythmic and non-rhythmic) concordant probe sets before (left) and after (right) bootstrapping for all pairs of normalization procedures, using the 10754 probe sets declared as rhythmic by RAIN under at least one normalization procedure in the mouse pituitary dataset.

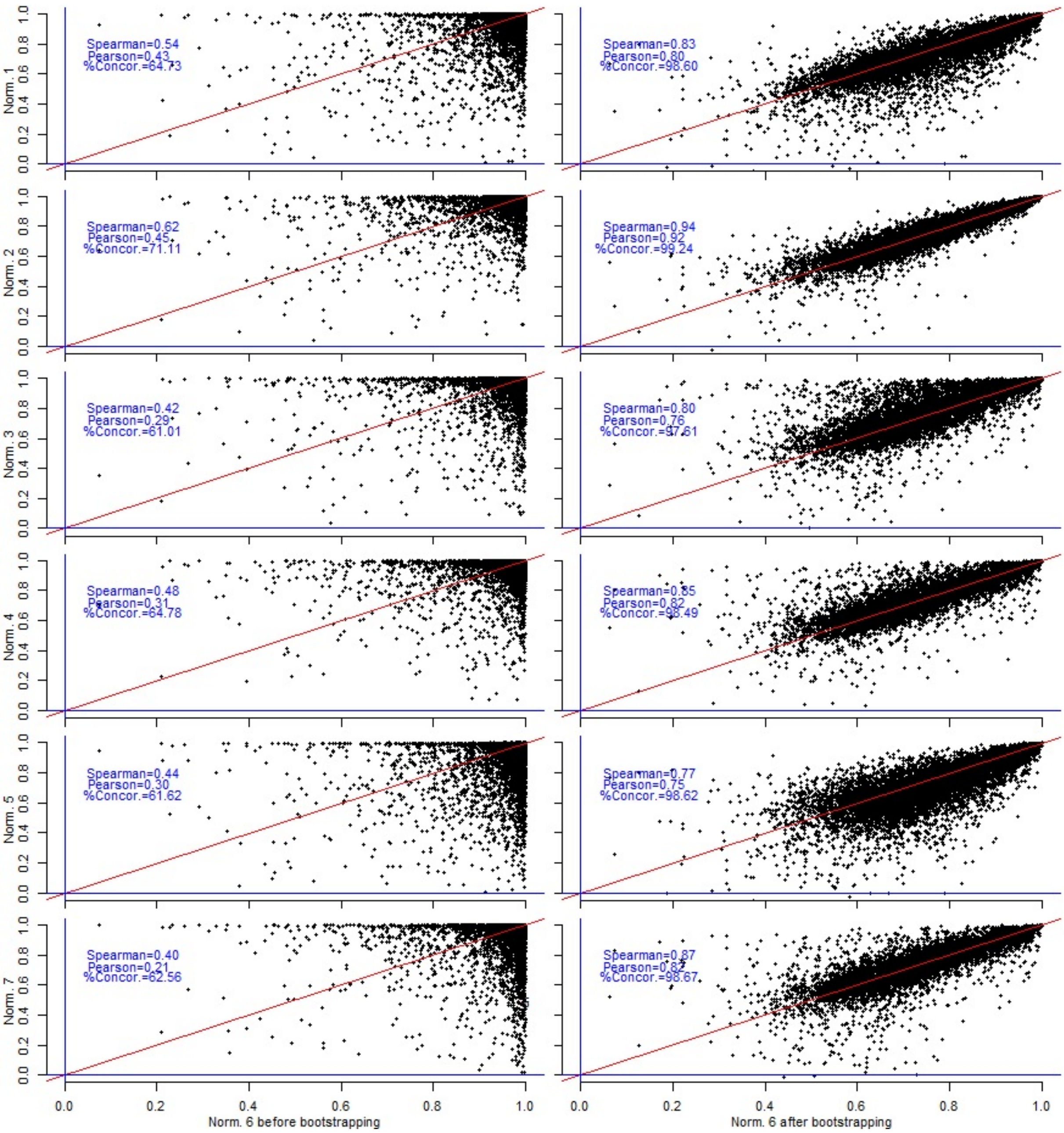

Figure S19: For each normalization method n, the left panels represent the pairwise scatter plots of $(M^g(n, ORIOS), M^g(Qspline, ORIOS))$ and the right panels represent the pairwise scatter plots of $(M^g_{Robust}(n, ORIOS), M^g_{Robust}(Qspline, ORIOS))$ derived from mouse pituitary. Red line is the 45° diagonal and the blue lines are the Cartesian axes.

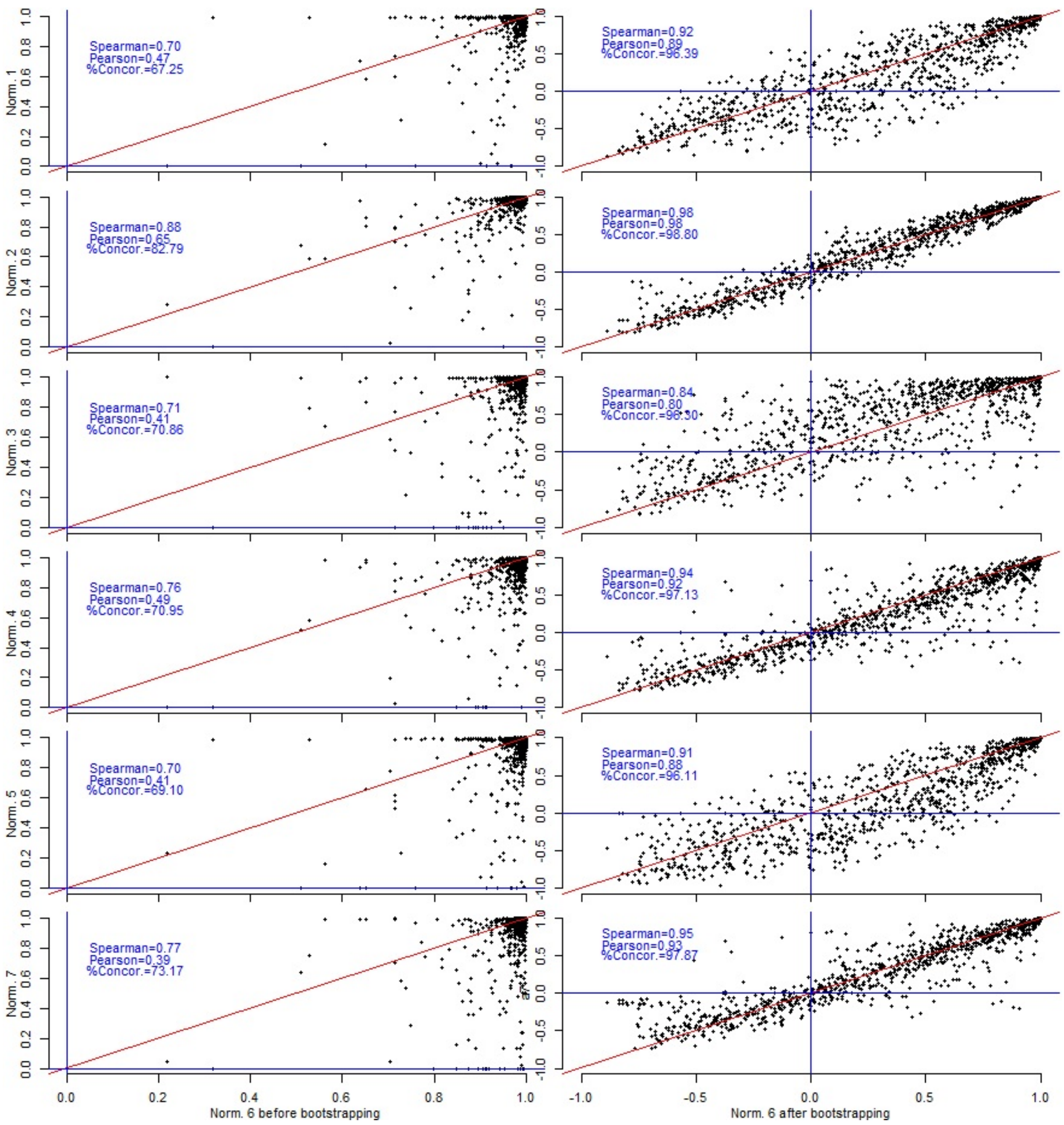

Figure S20: For each normalization method n, the left panels represent the pairwise scatter plots of $(M^g(n, JTK), M^g(Qspline, JTK))$ and the right panels represent the pairwise scatter plots of $(M^g_{Robust}(n, JTK), M^g_{Robust}(Qspline, JTK))$ derived from mouse pituitary. Red line is the 45° diagonal and the blue lines are the Cartesian axes.

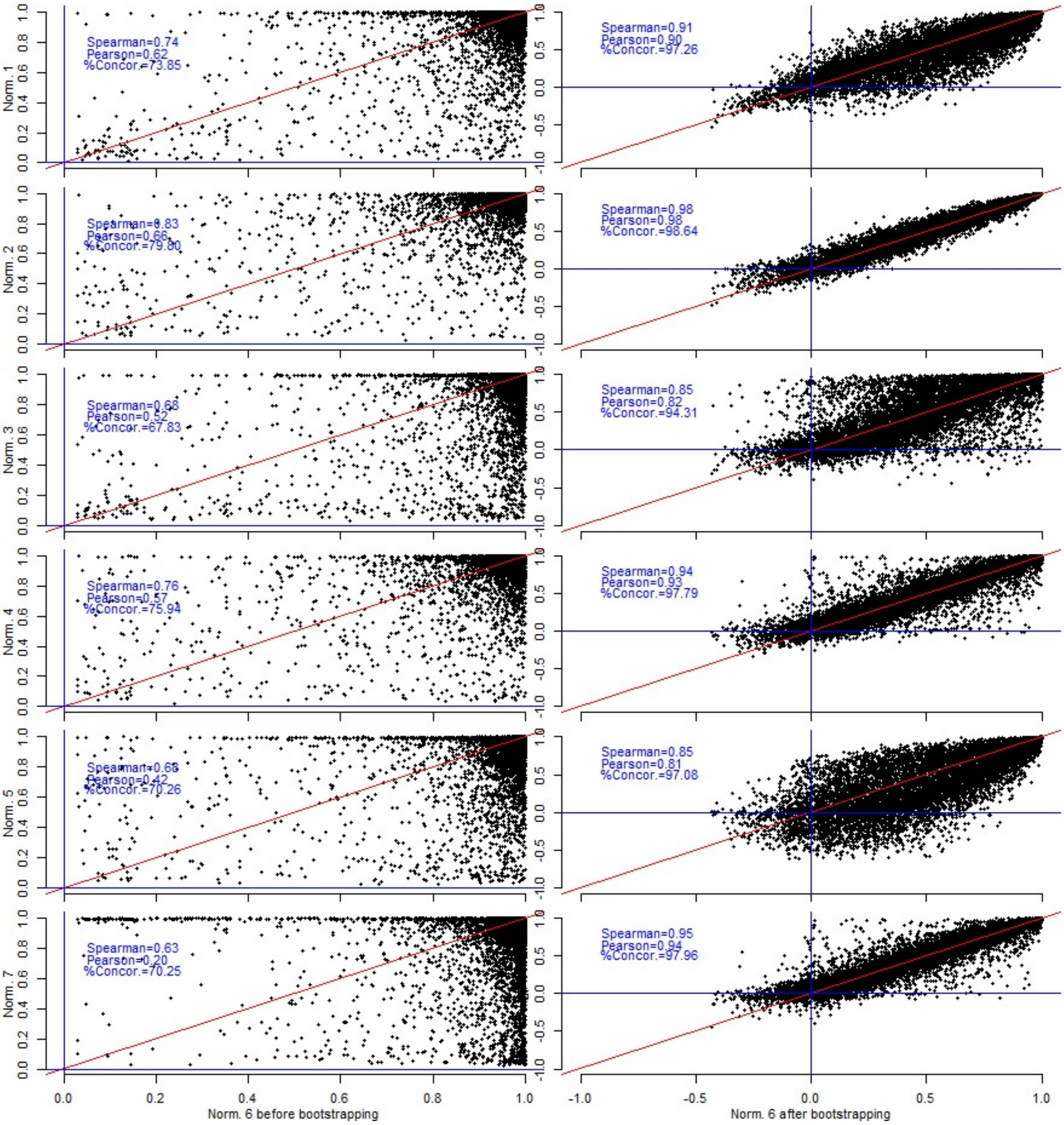

Figure S21: For each normalization method n, the left panels represent the pairwise scatter plots of $(M^g(n, RAIN), M^g(Qspline, RAIN))$ and the right panels represent the pairwise scatter plots of $(M^g_{Robust}(n, RAIN), M^g_{Robust}(Qspline, RAIN))$ derived from mouse pituitary. Red line is the 45° diagonal and the blue lines are the Cartesian axes.

2.3 Mouse NIH3T3 cell lines

Results derived from NIH3T3 cell lines dataset are similar to the obtained from liver and pituitary mouse tissues. Correlation and concordance analyses were limited to 3743, 117 and 9097 probe sets that were declared as rhythmic by at least one of the normalization methods according to ORIOS, JTK and RAIN algorithms respectively, see Table S2 for details. Similarly, correlation and concordance percentages substantially increase after bootstrapping, see Figures S22, S23 and S24 for ORIOS algorithm; Figures S25, S26 and S27 for JTK and Figures S28, S29 and S30 for RAIN. Figures S31, S32 and S33 show a more elliptic shape after considering bootstrap methodology for ORIOS, JTK and RAIN respectively and similar to the liver and the pituitary tissues JTK and RAIN results present higher uncertainty and variability.

Table S2. Number of genes in NIH3T3 cell lines originally detected as rhythmic by ORIOS, JTK and RAIN according to the different normalization strategies and $M^g(n, a) \geq 0.99$ for $g = 1, \ldots, 45101$

Normalization Strategy	ORIOS	JTK	RAIN
1 Quantile	1424	47	4778
2 Loess	1373	75	5051
3 Contrast	1370	72	5059
4 Constant	1709	54	4845
5 Invariant set	1010	47	4475
6 Qspline	1478	63	4793
7 VSN	1562	83	4899

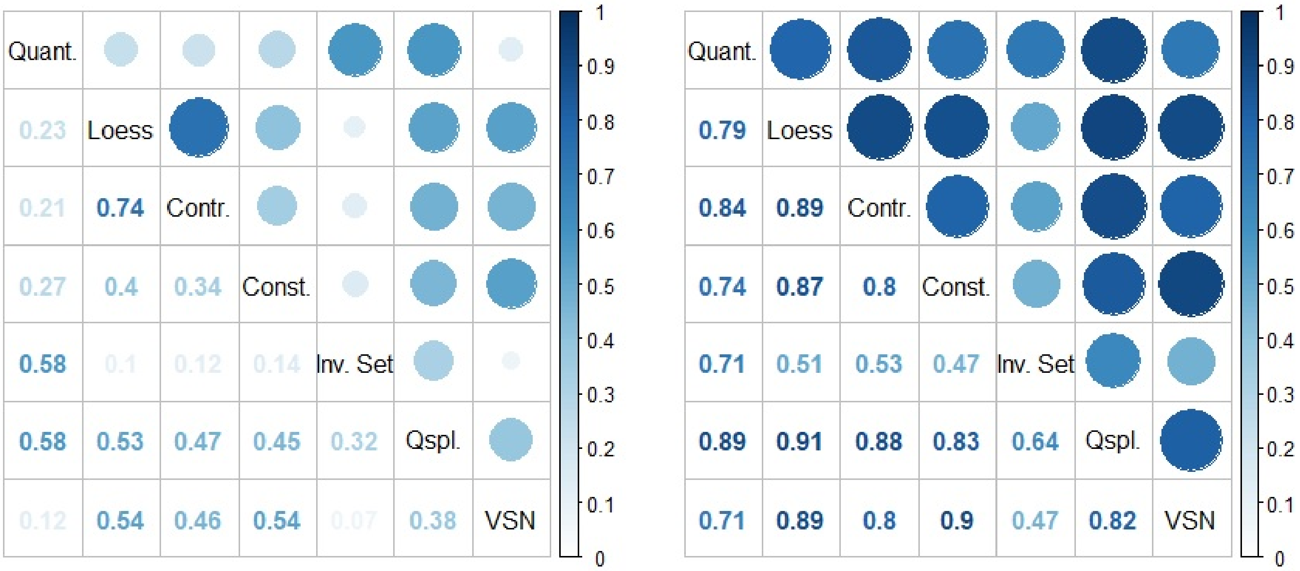

Figure S22: Spearman rank correlation coefficients between all pairs of normalization procedures considering the standard measure of rhythmicity (left) and the proposed robust measure (right) for the ORIOS algorithm, using the 3743 probe sets declared as rhythmic by ORIOS under at least one normalization procedure in the NIH3T3 cell lines dataset.

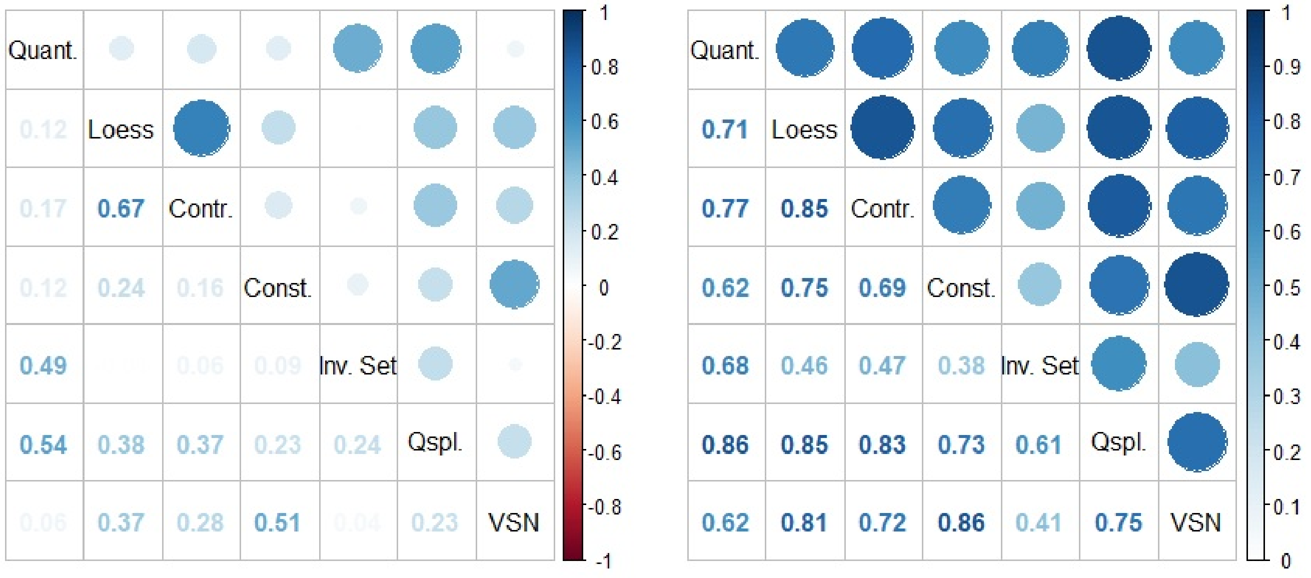

Figure S23: Pearson correlation coefficients between all pairs of normalization procedures considering the standard measure of rhythmicity (left) and the proposed robust measure (right) for the ORIOS algorithm, using the 3743 probe sets declared as rhythmic by ORIOS under at least one normalization procedure in the NIH3T3 cell lines dataset.

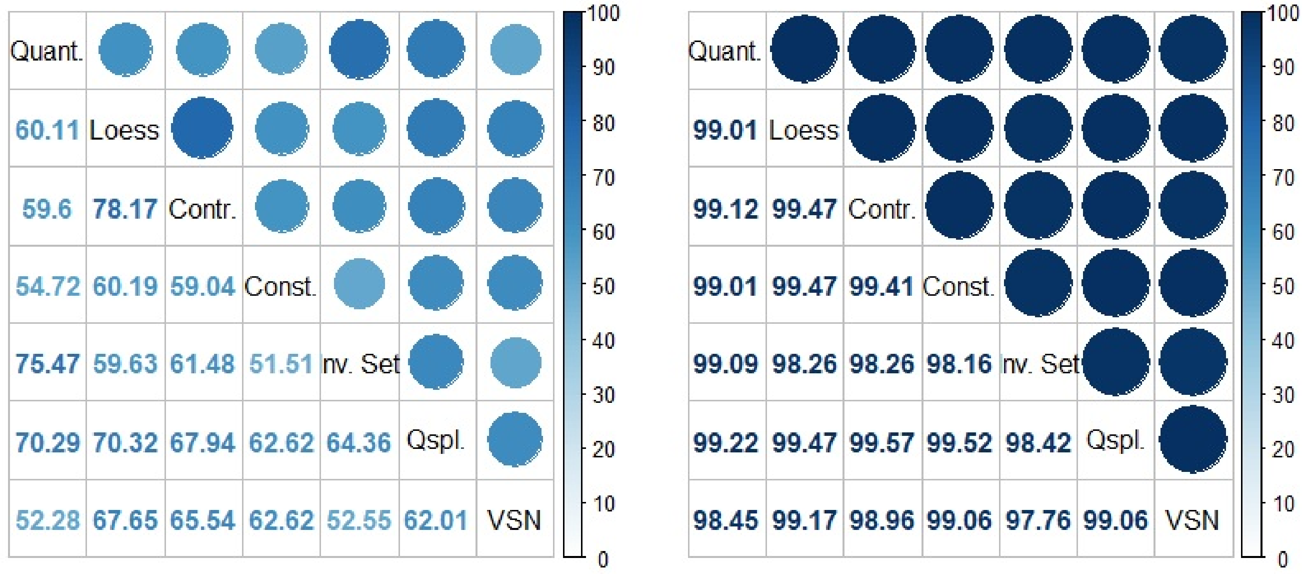

Figure S24: Percentage of (rhythmic and non-rhythmic) concordant probe sets before (left) and after (right) bootstrapping for all pairs of normalization procedures, using the 3743 probe sets declared as rhythmic by ORIOS under at least one normalization procedure in the NIH3T3 cell lines dataset.

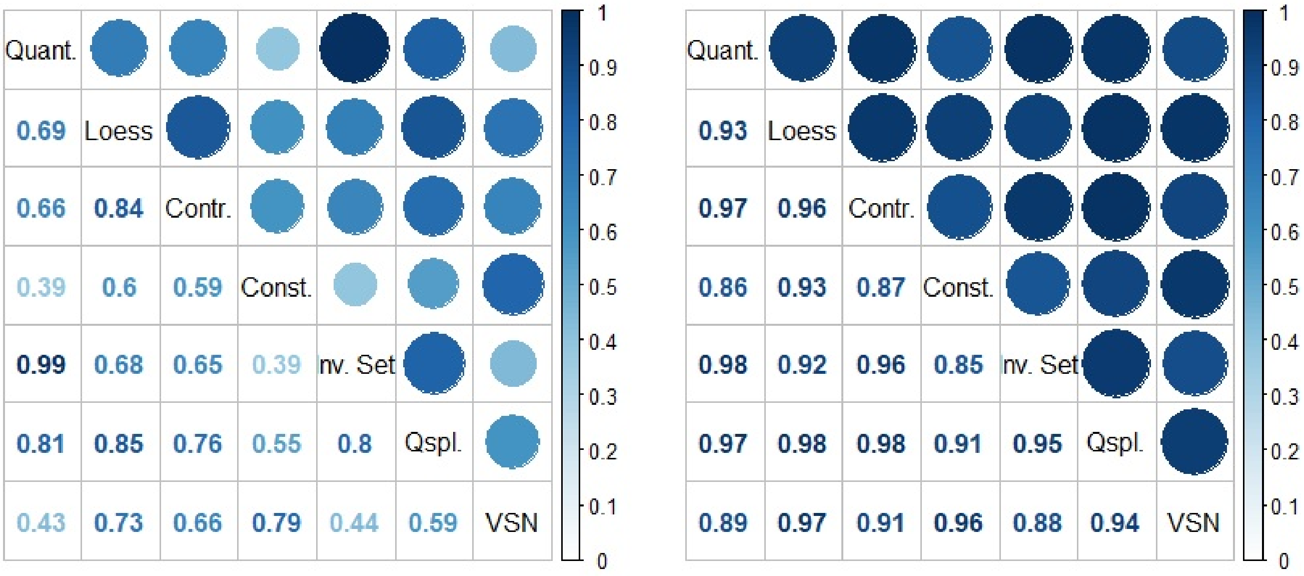

Figure S25: Spearman rank correlation coefficients between all pairs of normalization procedures considering the standard measure of rhythmicity (left) and the proposed robust measure (right) for the JTK algorithm, using the 117 probe sets declared as rhythmic by JTK under at least one normalization procedure in the NIH3T3 cell lines dataset.

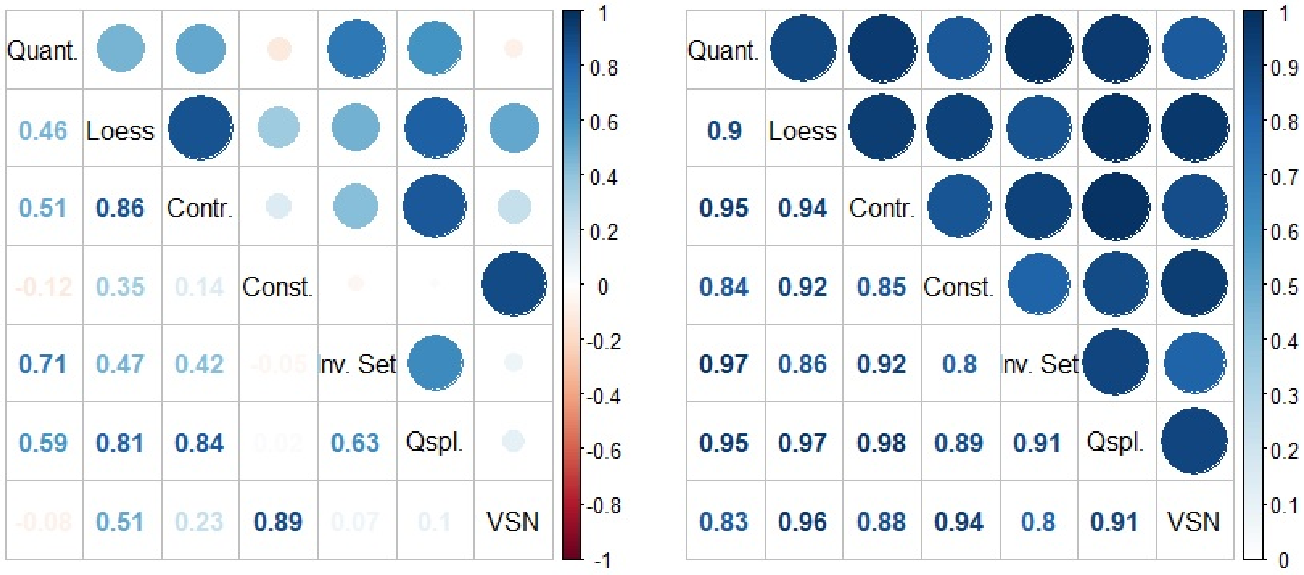

Figure S26: Pearson correlation coefficients between all pairs of normalization procedures considering the standard measure of rhythmicity (left) and the proposed robust measure (right) for the JTK algorithm, using the 117 probe sets declared as rhythmic by JTK under at least one normalization procedure in the NIH3T3 cell lines dataset.

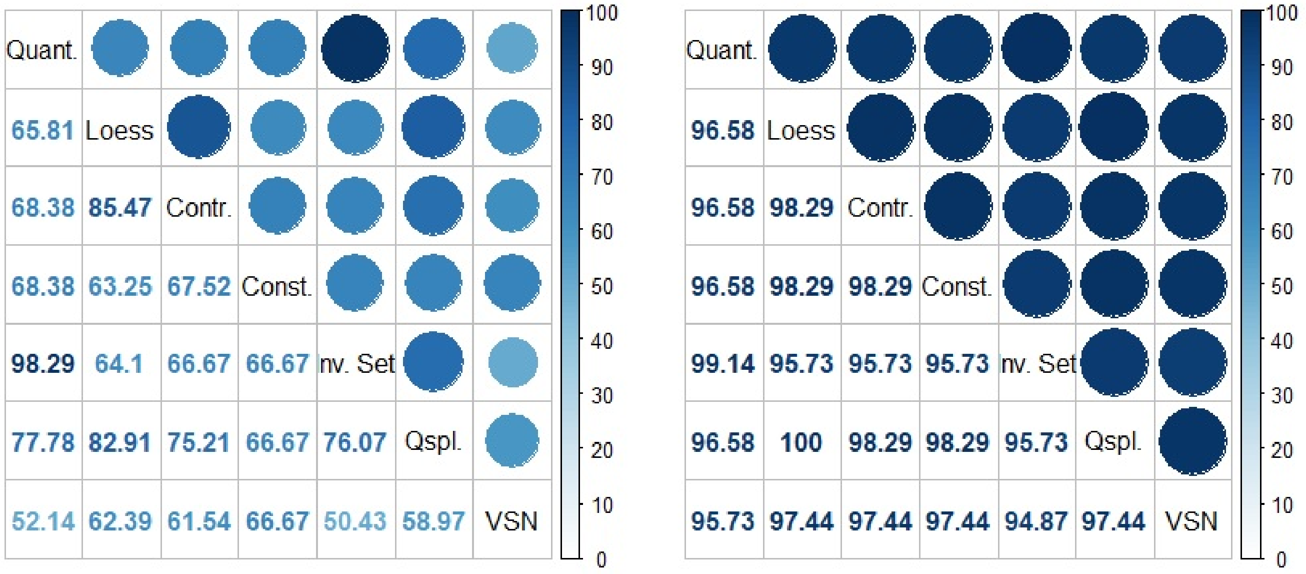

Figure S27: Percentage of (rhythmic and non-rhythmic) concordant probe sets before (left) and after (right) bootstrapping for all pairs of normalization procedures, using the 117 probe sets declared as rhythmic by JTK under at least one normalization procedure in the NIH3T3 cell lines dataset.

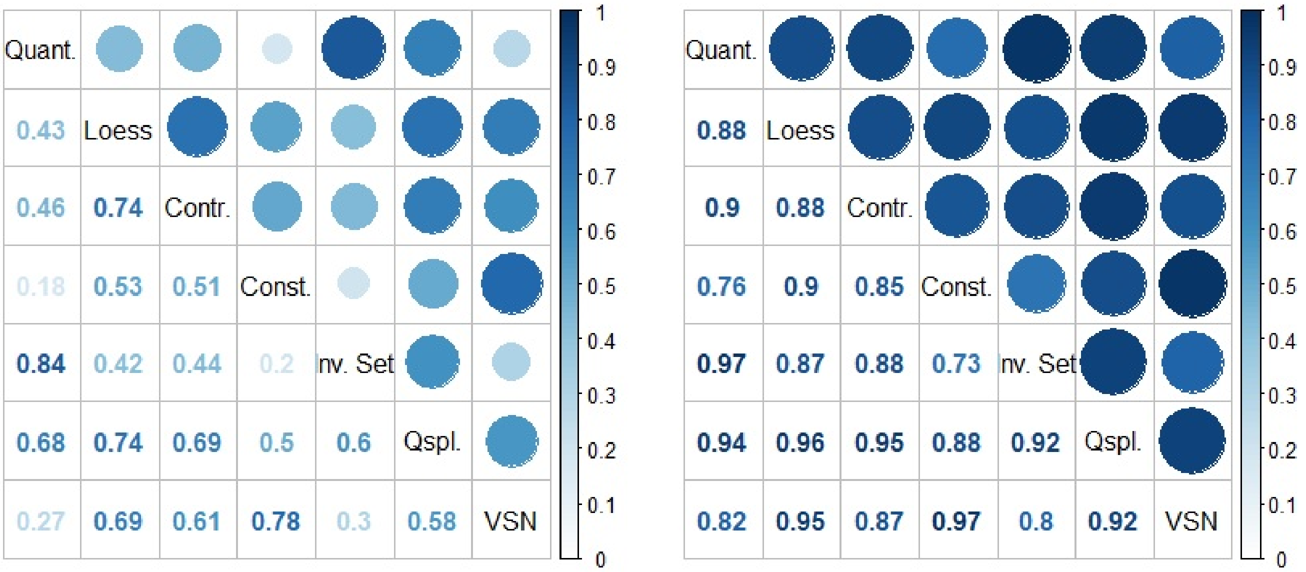

Figure S28: Spearman rank correlation coefficients between all pairs of normalization procedures considering the standard measure of rhythmicity (left) and the proposed robust measure (right) for the RAIN algorithm, using the 9097 probe sets declared as rhythmic by RAIN under at least one normalization procedure in the NIH3T3 cell lines dataset.

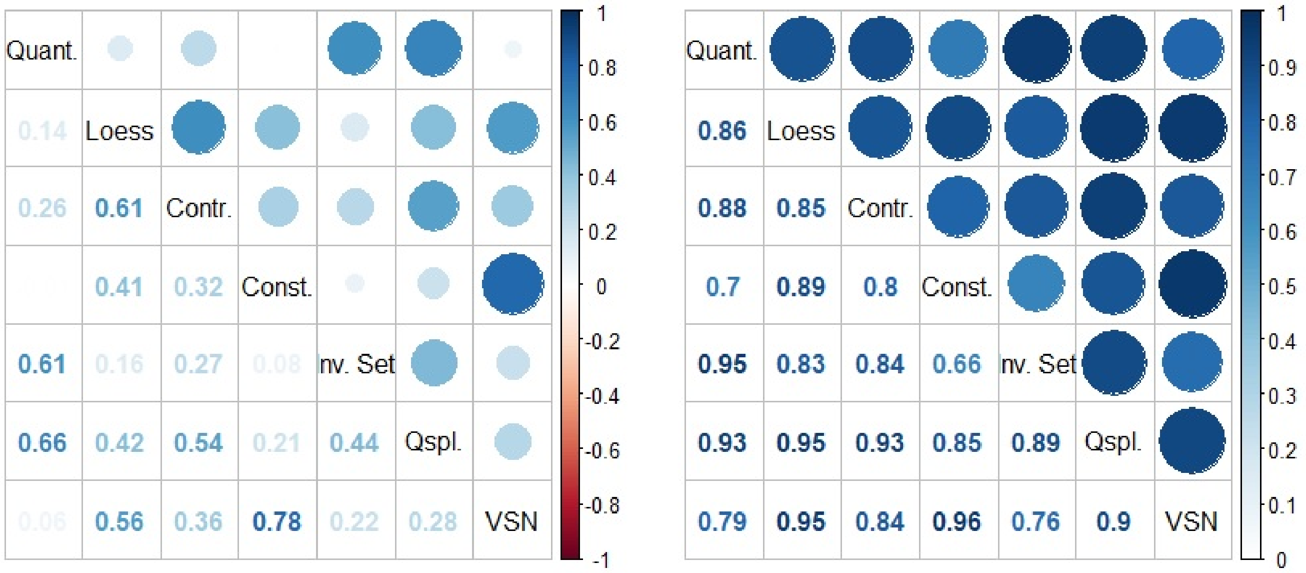

Figure S29: Pearson correlation coefficients between all pairs of normalization procedures considering the standard measure of rhythmicity (left) and the proposed robust measure (right) for the RAIN algorithm, using the 9097 probe sets declared as rhythmic by RAIN under at least one normalization procedure in the NIH3T3 cell lines dataset.

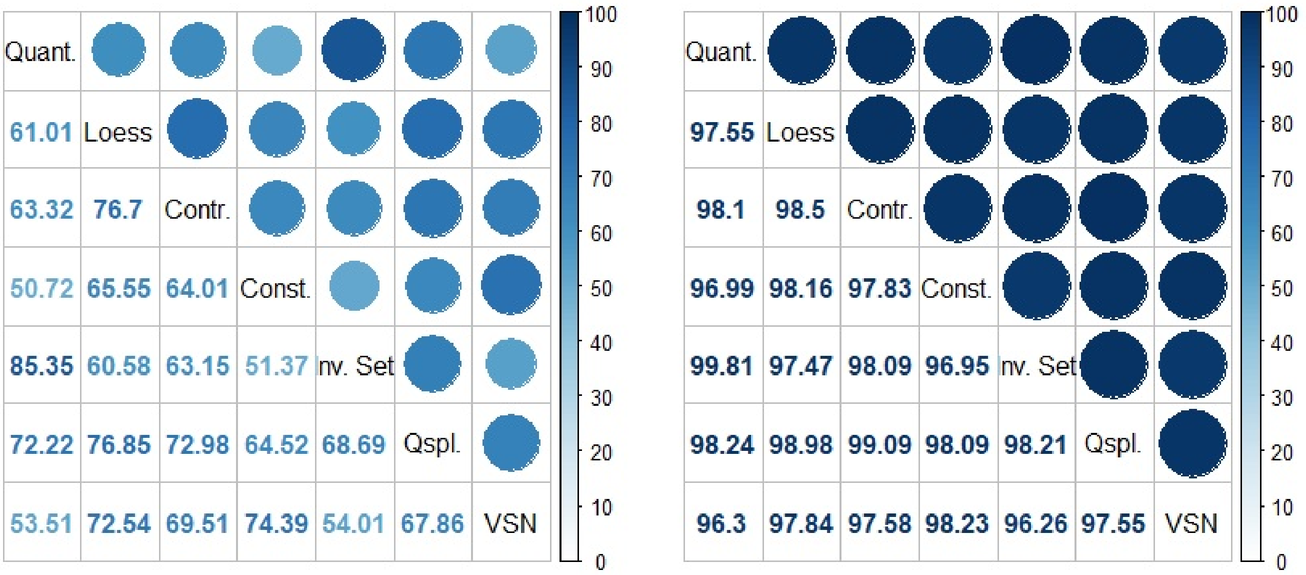

Figure S30: Percentage of (rhythmic and non-rhythmic) concordant probe sets before (left) and after (right) bootstrapping for all pairs of normalization procedures, using the 9097 probe sets declared as rhythmic by RAIN under at least one normalization procedure in the NIH3T3 cell lines dataset.

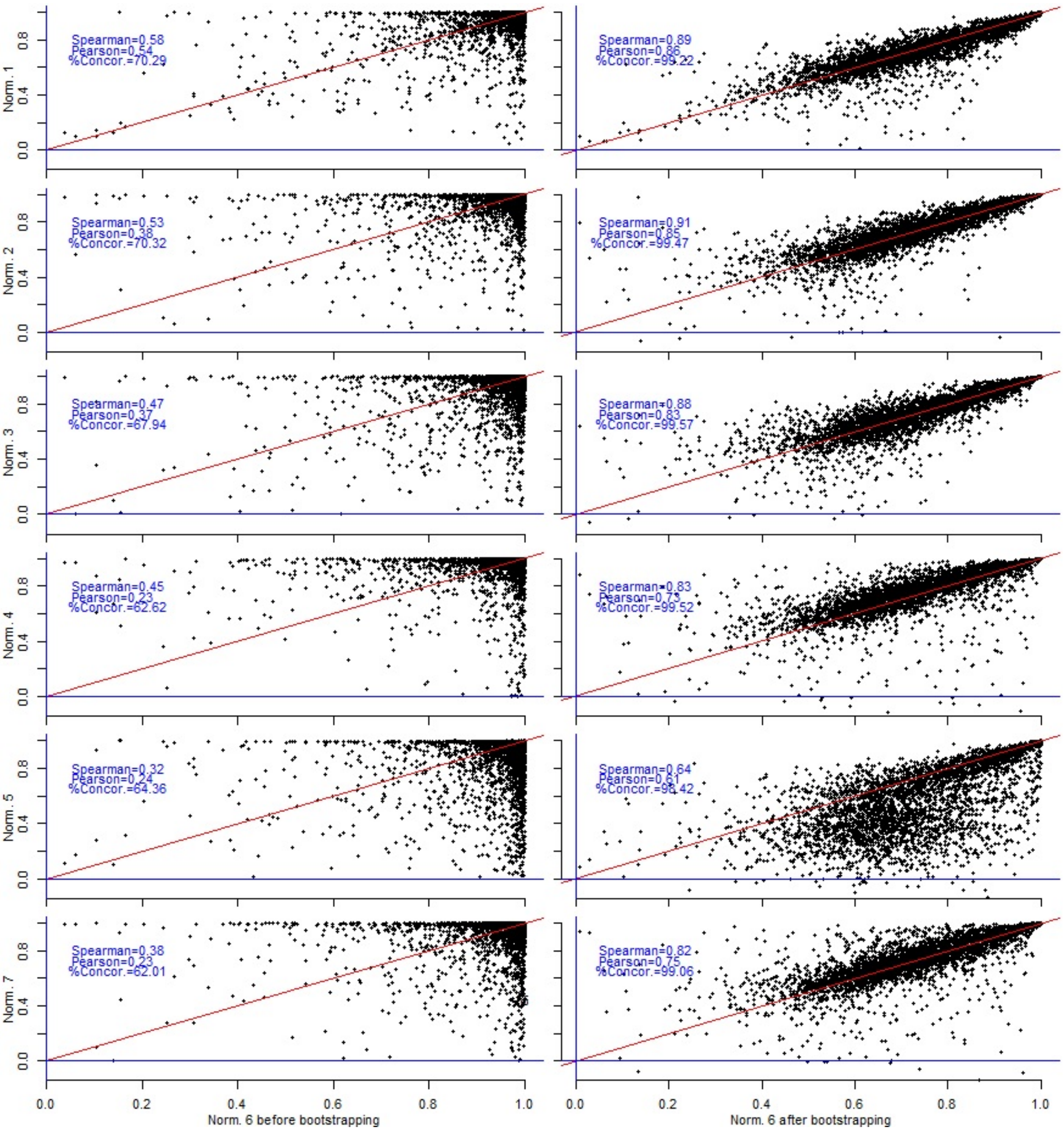

Figure S31: For each normalization method n, the left panels represent the pairwise scatter plots of $(M^g(n, ORIOS), M^g(Qspline, ORIOS))$ and the right panels represent the pairwise scatter plots of $(M^g_{Robust}(n, ORIOS), M^g_{Robust}(Qspline, ORIOS))$ derived from the NIH3T3 cell lines dataset. Red line is the 45° diagonal and the blue lines are the Cartesian axes.

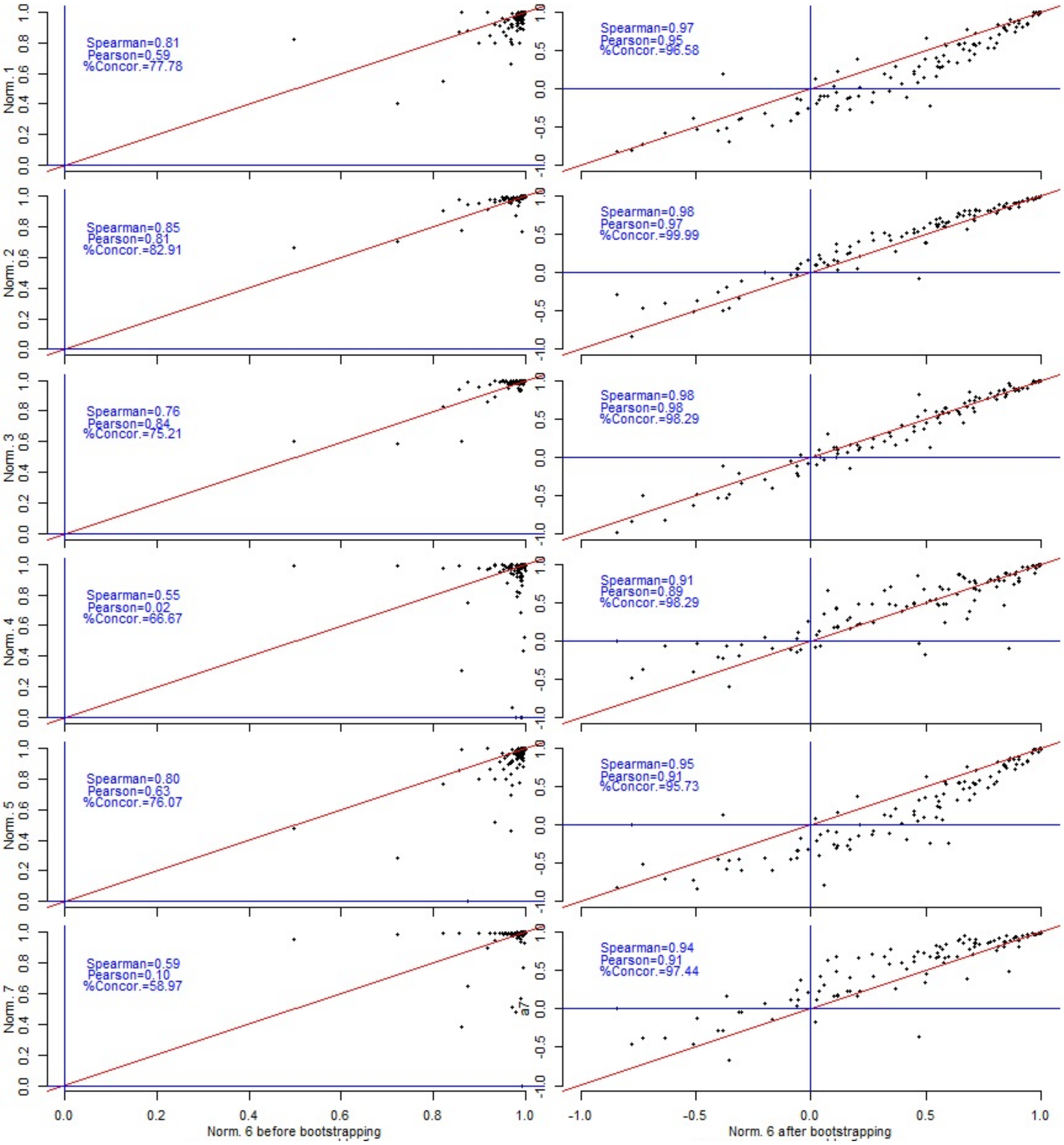

Figure S32: For each normalization method n, the left panels represent the pairwise scatter plots of $(M^g(n, JTK), M^g(Qspline, JTK))$ and the right panels represent the pairwise scatter plots of $(M^g_{Robust}(n, JTK), M^g_{Robust}(Qspline, JTK))$ derived from the NIH3T3 cell lines dataset. Red line is the 45° diagonal and the blue lines are the Cartesian axes.

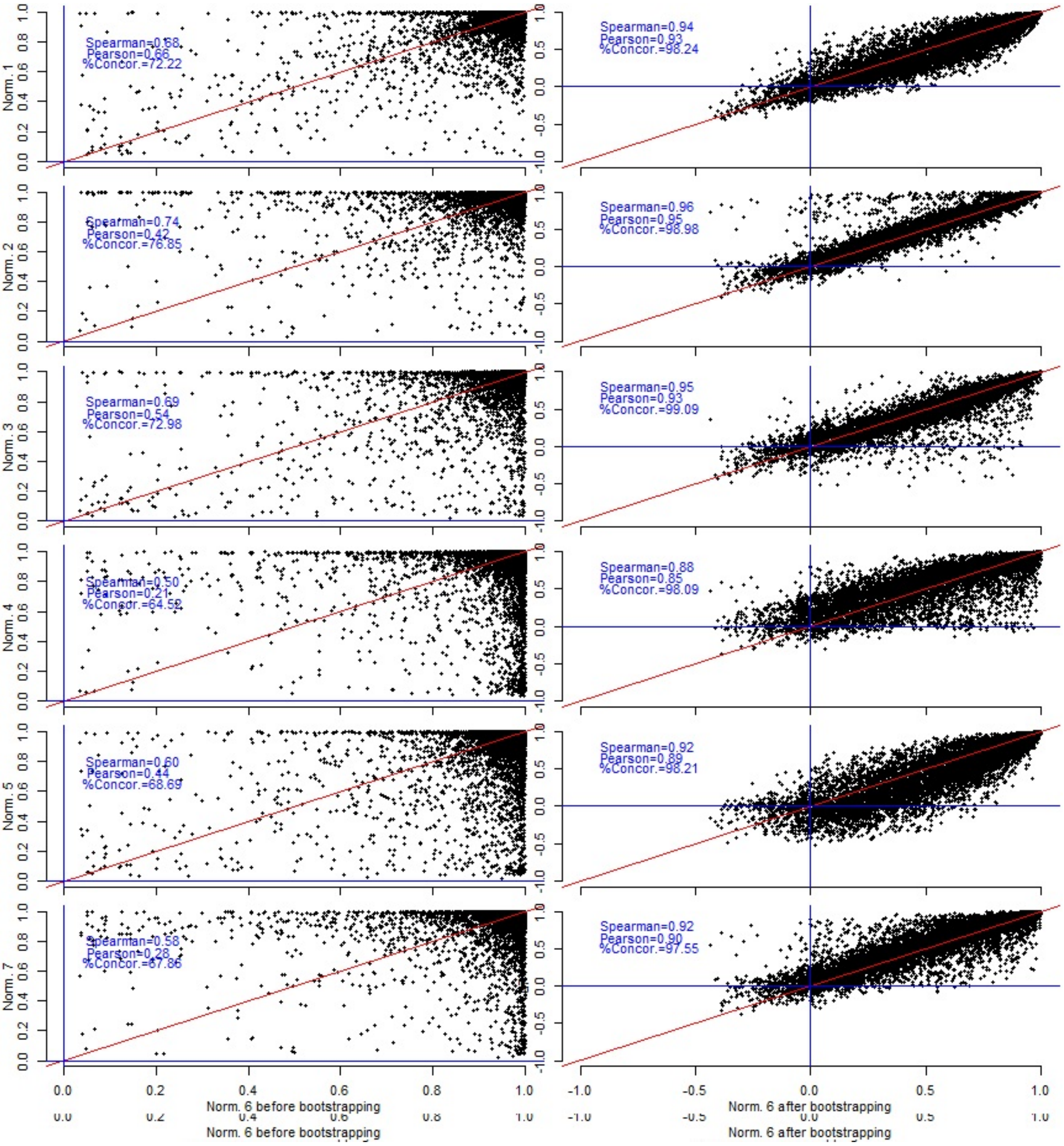

Figure S33: For each normalization method n, the left panels represent the pairwise scatter plots of $(M^g(n, RAIN), M^g(Qspline, RAIN))$ and the right panels represent the pairwise scatter plots of $(M^g_{Robust}(n, RAIN), M^g_{Robust}(Qspline, RAIN))$ derived from the NIH3T3 cell lines dataset. Red line is the 45° diagonal and the blue lines are the Cartesian axes.

REFERENCES

Emerson, J.D. and Hoaglin, D.C. (1983) *Analysis of two-way tables by medians. In Hoaglin, D.C., Mosteller, F. and Tukey, J.W. (eds) Understanding Robust and Exploratory Data Analysis.* New York: John Wiley & Sons, pp. 166-210.

Gautier, L., Cope, L., Bolstad, B.M. and Irizarry, R.A. (2004) Affy - Analysis of Affymetrix GeneChip data at the probe level. *Bioinformatics*. 20, 307-315.

Hughes, M.E., DiTacchio, L., Hayes, K.R., Vollmers, C., Pulivarthy, S., Baggs, J.E, Panda, S. and Hogenesch, J.B. (2009) Harmonics of circadian gene transcription in mammals. *PLoS Genet.* 5:e1000442. doi:10.1371/journal.pgen.1000442.

Hughes, M.E., Hogenesch, J.B. and Kornacker, K. (2010) JTK-CYCLE: An efficient nonparametric algorithm for detecting rhythmic components in genome-scale data sets. *J. Biol. Rhythm.* 25, 372-380.

Ihaka, R. and Gentleman, R. (1996) R: A language for data analysis and graphics. *J. Comput. Graph. Stat.* 5, 299-314.

Irizarry, R.A., Hobbs, B., Collin, F., Beazer-Barclay, Y.D., Antonellis, K.J., Scherf, U. and Speed, T.P. (2003) Exploration, normalization, and summaries of high density oligonucleotide array probe level data. *Biostatistics*. 4, 249-264. doi:10.1093/nar/gng015.

Larriba, Y., Rueda, C., Fernández, M.A. and Peddada, S.D. (2016) Order restricted inference for oscillatory systems for detecting rhythmic genes. *Nucleic Acids Res.* 44:e163. doi:10.1093/nar/gkw771.
